# Comparative Effectiveness of Midazolam, Propofol, and Dexmedetomidine in Patients With or at Risk for Acute Respiratory Distress Syndrome: A Propensity Score-Matched Cohort Study

**DOI:** 10.3389/fphar.2021.614465

**Published:** 2021-03-23

**Authors:** An-Min Hu, Xiong-Xiong Zhong, Zhen Li, Zhong-Jun Zhang, Hui-Ping Li

**Affiliations:** ^1^ Department of Anesthesiology, Shenzhen People’s Hospital, Shenzhen, China; ^2^ The Second Clinical Medical College, Jinan University, Shenzhen, China; ^3^ Department of Anesthesiology, First Affiliated Hospital, Southern University of Science and Technology, Shenzhen, China; ^4^ Shenzhen Research Institute of Big Data, Shenzhen, China; ^5^ Department of Critical Care Medicine, Shenzhen People’s Hospital, Shenzhen, China

**Keywords:** sedative agents, Acute Respiratory Distress Syndrome, intensive care unit care, mortality effects, comparative effectiveness research

## Abstract

**Background:** Sedatives are commonly used in patients with or at risk for acute respiratory distress syndrome (ARDS) during mechanical ventilation. To systematically compare the outcomes of sedation with midazolam, propofol, and dexmedetomidine in patients with or at risk for ARDS.

**Methods:** We developed a dataset of real-world data to enable the comparison of the effectiveness and safety of sedatives and the associated outcomes from the MIMIC-III database and the eICU Collaborative Research database. We performed a systematic study with six cohorts to estimate the relative risks of outcomes among patients administered different sedatives. Propensity score matching was performed to generate a balanced 1:1 matched cohort and to identify potential prognostic factors. The outcomes included hospital mortality, duration of mechanical ventilation, length of intensive care unit stay, length of hospitalization, and likelihood of being discharged home.

**Results:** We performed 60 calibrated analyses among all groups and outcomes with 17,410 eligible patients. Sedation with dexmedetomidine was associated with a lower in-hospital mortality rate than sedation with midazolam and propofol or sedation without dexmedetomidine (*p* < 0.001). When compared with no sedation, the use of midazolam, propofol or dexmedetomidine was associated with a longer ICU stay and longer hospitalization duration (*p* < 0.01). Patients treated with midazolam were relatively less likely to be discharged home (*p* < 0.05).

**Conclusion:** Patients treated with dexmedetomidine had a reduced risk of mortality. These data suggest that dexmedetomidine may be the preferred sedative in patients with or at risk for ARDS.

## Introduction

Acute respiratory distress syndrome (ARDS) is a clinical syndrome in critically ill patients involving acute respiratory failure and noncardiogenic pulmonary edema (Fan et al., 2018; Matthay et al., 2019). The administration of sedative drugs is a nearly universal intervention in mechanically ventilated intensive care unit (ICU) patients (Patel and Kress, 2012). Appropriate sedation management is one effective method of improving patient tolerance of mechanical ventilation and reducing psychological stress in critically ill patients in the ICU (Weinert and Calvin, 2007; Patel and Kress, 2012); however, the use of an inappropriate sedation strategy may increase the risk of all-cause mortality, delay ventilator weaning, and prolong the duration of hospitalization in ICU patients undergoing mechanical ventilation (Shehabi et al., 2012; Shehabi et al., 2013b; Shah et al., 2017; Shehabi et al., 2018; Aragón et al., 2019).

The 2013 Pain, Agitation, and Delirium (PAD) guidelines suggest that either propofol or dexmedetomidine may be preferable to midazolam in mechanically ventilated adult ICU patients because of the associated reduced duration of mechanical ventilation, ICU length of stay (LOS), and delirium (Barr et al., 2013). However, these recommendations are based on evidence from earlier clinical trials that compared two drugs; these studies lack both relevance and validity (Shehabi et al., 2013a). Thus, there remains uncertainty, and real-world evidence of the relative effectiveness of common sedatives in this patient population is lacking.

Accordingly, we evaluated multiple characteristics of ARDS (MFAS) to compare the use of midazolam, propofol, and dexmedetomidine in patients with or at risk for ARDS using data from open source ICU databases. MFAS provides a comprehensive comparison of the findings and their consistency across populations, hospital characteristics and outcomes.

## Methods

### Data Source

The Multiparameter Intelligent Monitoring in Intensive Care (MIMIC) III (version 1.4) database and eICU Collaborative Research Database are maintained by the Laboratory for Computational Physiology at Massachusetts Institute of Technology (Johnson et al., 2016; Pollard et al., 2018). The databases are accessible to researchers who have passed training courses on protecting human subjects. Data were extracted by author AMH (certification number: 26,450,451).

### Study Population and Stratification

Mechanically ventilated ICU patients with a diagnosis of ARDS or at risk for ARDS were included. ARDS risk factors included acute hypoxemic respiratory failure, pneumonia, sepsis, trauma, burns, and other diagnoses or treatments (i.e., multiple transfusions) (Gajic et al., 2011; Bellani et al., 2016; Thompson et al., 2017; Auriemma et al., 2020).

Patient diagnoses were determined based on the International Classification of Disease, Ninth Revision, Clinical Modification (ICD-9-CM) (Supplementary Material S1) (Sottile et al., 2018). Comorbidities were recorded according to the Elixhauser Comorbidity Index based on the diagnoses recorded during hospitalization (Quan et al., 2005).

The inclusion criteria in this study were as follows: 1) for patients with multiple ICU stays, only the first ICU stay was eligible; 2) adults ≥18 years of age on ICU admission; 3) ICU stay ≥24 h; and 4) the use of invasive mechanical ventilation (MV) for at least 12 h.

Patients were enrolled into six cohorts: midazolam vs. no midazolam, propofol vs. no propofol, dexmedetomidine vs. no dexmedetomidine, midazolam vs. propofol, midazolam vs. dexmedetomidine, propofol vs. dexmedetomidine.

### Outcomes

The following outcome measures were determined: hospital mortality, days on ventilation, ICU LOS, hospitalization duration, and discharge destination (home vs. elsewhere).

### Data Analysis

Patient characteristics included age; sex; ethnicity; weight; height; acute physiology and chronic health evaluation (APACHE) III score; oxygenation index; alveolar-arterial oxygen difference (AaDo2); patient characteristics at discharge; hospital characteristics; and the use of sedatives or opioids. The APACHE-III scoring system is designed to prospectively predict mortality in individual ICU patients (Knaus et al., 1991). We ensured that the diagnosis of ARDS or a known risk factor for ARDS was present at the time of admission. Patient characteristics at discharge included the following: ARDS, pneumonia, sepsis, aspiration, heart failure, chronic obstructive pulmonary disease (COPD), disseminated intravascular coagulation (DIC), liver disease, renal failure, hypertension, and diabetes. Hospital characteristics were defined according to the database, including ICU type, number of ICU beds, teaching status, and provider region. Sedatives or opioids included the following: midazolam, propofol, dexmedetomidine, fentanyl, or none.

Descriptive data are presented as the medians (25th to 75th percentiles) for continuous variables and frequencies (%) for categorical variables. Categorical variables were compared between groups using the chi-square test. Unpaired t-tests or Kruskal-Wallis tests were used for continuous variables.

Propensity score generation, stratification by deciles, and 1:1 matching between groups were performed using the R package MatchIt (Ho et al., 2011). A nonparsimonious regression model was used to produce a propensity score for the group with fewer patients using the patient characteristics described above. For the propensity-score analysis (our primary analysis), each patient in the group with fewer patients was matched to their nearest neighbor within 0.001. We chose to match to the third decimal point (a caliper of 0.001) because this value is less than 0.02 SDs of the propensity score, which is a commonly suggested range. Estimating the propensity score using a logit model resulted in both reasonable matches and the right overall sample size. The final models included the hospital as a random effect and all patient characteristics used to calculate the propensity score. Additionally, multivariable regression modeling, including all the patient characteristics used to calculate the propensity score, was performed to confirm these findings (secondary analysis).

The following pre-specified subgroups and interactions were assessed: age (within 18–65, and 65 years or older), duration of MV (within 12–24, within 24–48, and 48 h or longer).

Missing data were imputed with the Multivariate Imputation by Chained Equations (MICE) method (Buuren and Groothuis-Oudshoorn, 2011). The amount of missing data was low and was detailed in Supplementary Material S2. Supplementary Material S3 shows the frequency of missing data elements and the distribution of each parameter before and after imputation. All analyses were performed using R version 3.62.

## Results

This study was conducted and reported in accordance with strengthening the reporting of observational studies in epidemiology (STROBE) guidelines (Supplementary Material S4) (Von Elm et al., 2007). In addition to the 46,428 ICU patients and 61,051 ICU admissions in the MIMIC-III database v1.4, 177,863 ICU patients and 626,858 ICU admissions in the eICU Collaborative Research database were available. Sequentially, we excluded 8,433 patients whose age at admission was younger than 18 years, 116,599 patients who stayed in the ICU for less than 24 h, and 81,849 patients who received mechanical ventilation for less than 12 h, as shown in Supplementary Material S5. The final 17,410 patients had at least one ARDS risk factor: 12,567 patients had acute hypoxemic respiratory failure, 4,517 patients had pneumonia, 4,757 patients had sepsis, and 1,603 patients had experienced aspiration.

Table 1 summarizes the characteristics of the studied subjects. A total of 2,719 patients (15.6%) were sedated with midazolam, 7,559 (43.4%) with propofol and 2,234 (12.8%) with dexmedetomidine. Before propensity score matching, there were statistically significant differences in admission type in the stratified analyses between midazolam and no midazolam, propofol and no propofol, dexmedetomidine and no dexmedetomidine, midazolam and propofol, midazolam and dexmedetomidine, and propofol and dexmedetomidine. Overall, patients who received midazolam, propofol or dexmedetomidine were older, weighed more, had a lower oxygenation index, had a higher AaDo2, and were more likely to be white, have ARDS, or have experienced aspiration than patients who did not receive those agents (Supplementary Materials S6–S8). Patients receiving midazolam had a higher oxygenation index, had a lower AaDo2 and were less likely to have ARDS, pneumonia, sepsis, aspiration, chronic pulmonary disease, liver disease, or renal disease than patients receiving propofol or dexmedetomidine (Supplementary Materials S9, S10). We found that new dexmedetomidine users were more likely to be female, had a lower oxygenation index, and were more likely to have previously used midazolam than propofol users (Supplementary Material S11). However, propensity score matching led to an adequate balance among the groups with respect to the covariates, reducing concerns that the measured effects were due to baseline confounders.

**TABLE 1:** Baseline characteristics of the patients.

Clinical variable^a^	All subjects (*N* = 17,410)	Midazolam (*N* = 2,719)	Propofol (*N* = 7,559)	Dexmedetomidine (*N* = 2,234)
Age, no. (%)				
18–45 years	2,452 (14.1)	504 (18.5)	1,225 (16.2)	367 (16.4)
46–65 years	6,601 (37.9)	1,146 (42.1)	3,002 (39.7)	892 (39.9)
66–80 years	5,859 (33.7)	797 (29.3)	2,411 (31.9)	736 (32.9)
81–89 years	2067 (11.9)	229 (8.4)	787 (10.4)	208 (9.3)
Over 89 years	431 (2.5)	43 (1.6)	134 (1.8)	31 (1.4)
**Female, no. (%)**	7,679 (44.1)	1,123 (41.3)	3,261 (43.1)	887 (39.7)
**Ethnicity, no. (%)**				
White	12,992 (74.6)	2,101 (77.3)	5,699 (75.4)	1,629 (72.9)
Black	2,112 (12.1)	240 (8.8)	682 (9.0)	192 (8.6)
Latino	997 (5.7)	153 (5.6)	578 (7.6)	246 (11.0)
Asian	229 (1.3)	32 (1.2)	88 (1.2)	16 (0.7)
Other	1,080 (6.2)	193 (7.1)	512 (6.8)	151 (6.8)
**Weight, kg**	80.3 (66.7–98.8)	83.1 (68.0–100.0)	81.6 (68–99.6)	81.6 (68.0–99.0)
**Height, cm**	170.1 (162.6–177.8)	170.2 (162.6–177.8)	170.2 (162.6–177.8)	170.2 (162.6–177.8)
**Apache-III score**	53 (37–73)	61 (42–83)	54 (38–74)	54 (39–73)
**Oxgenation index, mmHg**	166.4 (104.4–218)	129.0 (68.0–190.0)	162.4 (97.9–217.5)	144.0 (83.0–196.6)
**AaDo2, mmHg**	293.9 (208.1–471.2)	361.6 (241.0–562.6)	305.0 (213.6–489.1)	332.4 (216.6–540.0)
**Patient characteristics at discharge, no. (%)**				
ARDS	12,567 (72.2)	2,226 (81.9)	5,769 (76.3)	1715 (76.8)
Pneumonia	4,517 (25.9)	1,043 (38.4)	1894 (25.1)	614 (27.5)
Sepsis	4,757 (27.3)	941 (34.6)	1965 (26.0)	530 (23.7)
Aspiration	1,603 (9.2)	396 (14.6)	817 (10.8)	267 (12.0)
Heart failure	1,014 (5.8)	220 (8.1)	504 (6.7)	126 (5.6)
Chronic pulmonary disease	2,447 (14.1)	445 (16.4)	1,051 (13.9)	287 (12.8)
Liver disease	375 (2.2)	106 (3.9)	150 (2.0)	31 (1.4)
Renal failure	2057 (11.8)	315 (11.6)	890 (11.8)	184 (8.2)
Hypertension	2,890 (16.6)	535 (19.7)	1,441 (19.1)	372 (16.7)
Diabetes	576 (3.3)	174 (6.4)	295 (3.9)	103 (4.6)
**Hospital characteristics**				
**ICU Type, no. (%)**				
SICU	1,699 (9.8)	360 (13.2)	706 (9.3)	165 (7.4)
CCU	3,395 (19.5)	503 (18.5)	1,405 (18.6)	468 (20.9)
NICU	1,008 (5.8)	107 (3.9)	371 (4.9)	132 (5.9)
Others	11,308 (65)	1749 (64.3)	5,077 (67.2)	1,469 (65.8)
**Number of beds, no. (%)**				
<100	339 (1.9)	19 (0.7)	66 (0.9)	3 (0.1)
100–249	3,533 (20.3)	470 (17.3)	1,628 (21.5)	377 (16.9)
250–499	4,749 (27.3)	509 (18.7)	2,501 (33.1)	530 (23.7)
≥500	8,789 (50.5)	1721 (63.3)	3,364 (44.5)	1,324 (59.3)
**Teaching, no. (%)**	5,556 (31.9)	1,334 (49.1)	1772 (23.4)	522 (23.4)
**Provider region, no. (%)**				
Midwest	5,501 (31.6)	587 (21.6)	2034 (26.9)	877 (39.3)
Northeast	2,844 (16.3)	1,168 (43)	1,199 (15.9)	191 (8.5)
South	5,821 (33.4)	705 (25.9)	2,309 (30.5)	886 (39.7)
West	3,244 (18.6)	259 (9.5)	2017 (26.7)	280 (12.5)
**Using first-line sedation or opioids drugs, no. (%)**				
Midazolam	2,719 (15.6)	2,719 (100.0)	1,470 (19.4)	668 (29.9)
Propofol	7,559 (43.4)	1,470 (54.1)	7,559 (100.0)	1,510 (67.6)
Dexmedetomidine	2,234 (12.8)	668 (24.6)	1,510 (20)	2,234 (100.0)
Morphine	601 (3.5)	261 (9.6)	393 (5.2)	101 (4.5)
Fentanyl	5,515 (31.7)	2,102 (77.3)	3,606 (47.7)	1,173 (52.5)
Not using the four drugs	7,203 (41.4)	775 (28.5)	2,834 (37.5)	876 (39.2)

**Abbreviations:** AaDO2, alveolar-arterial oxygen difference; APACHE-III score, the acute physiology and chronic health evaluation III score; CCU, cardiac care unit; NICU, neurological intensive care unit; SICU, surgical intensive care unit.

^a^ Data shown as mean ± standard deviation, number (percent), or median (interquartile range) as appropriate.

### Propensity-Matched Analysis

The fully adjusted, propensity score-matched analysis for outcomes is shown in Table 2. There was no difference in the mortality rate between patients who were sedated with midazolam and those who were not sedated with midazolam or between those who were sedated with midazolam and those who were sedated with propofol; however, patients who were sedated with midazolam had a higher mortality rate than patients who were sedated with dexmedetomidine. Patients who were sedated with propofol had a lower mortality rate than patients who were sedated without propofol and, surprisingly, a higher mortality rate than patients who were sedated with dexmedetomidine. Moreover, the mortality rate was significantly reduced for those sedated with dexmedetomidine.

**TABLE 2:** Results of propensity-matched analysis in patients with or at risk for acute respiratory distress syndrome.

	Comparator	Hospital mortality^a^	Ventilator days^b^	ICU days^b^	Hospital days^b^	Discharge to home^a^
Midazolam	No Midazolam	1.01 (0.86–1.21), 0.86	0.04 (−0.14–0.22), 0.69	3.25 (2.60–3.91), <0.001	2.21 (0.74–3.68), <0.01	0.78 (0.67–0.91), <0.01
Midazolam	Propofol	1.23 (0.96–1.57), 0.10	0.11 (−0.14–0.35), 0.38	−0.09 (−0.87–0.69), 0.82	0.45 (−0.89–1.79), 0.51	0.77 (0.62–0.95), 0.02
Midazolam	Dex	1.92 (1.48–2.51), <0.001	−0.03 (−0.28–0.21), 0.81	−0.22 (−0.88–0.44), 0.51	−0.66 (−2.33–0.99), 0.43	0.80 (0.64–0.99), 0.04
Propofol	No Propofol	0.69 (0.59–0.81), <0.001	0.11 (−0.05–0.27), 0.18	3.33 (2.77–3.88), <0.001	3.29 (2.18–4.39), <0.001	1.06 (0.92–1.22), 0.39
Propofol	Dex	1.72 (1.26–2.35), <0.001	−0.16 (−0.39–0.06), 0.15	0.17 (−0.55–0.89), 0.65	−0.66 (−3.02–1.70), 0.58	0.87 (0.68–1.10), 0.24
Dex	No Dex	0.44 (0.37–0.52), <0.001	0.30 (0.14–0.46), <0.01	2.37 (1.88–2.85), <0.001	2.94 (1.95–3.93), <0.001	1.03 (0.90–1.18), 0.66

**Abbreviation:** Dex, dexmedetomidine.

^a^ Data are presented as odds ratio (95% confidence interval), *p* value.

^b^ Data are presented as difference of variable value (95% confidence interval), *p* value.

Patients who took midazolam had no difference in ventilation time compared with patient who did not take midazolam, patients who were sedated with propofol and those who were sedated with dexmedetomidine (Table 2). Patients who were sedated with midazolam had longer ICU stays than those who were not sedated with midazolam but no difference in the LOS in the ICU when compared with patients sedated with propofol and dexmedetomidine. Similarly, patients sedated with midazolam had a longer hospital stay than those who were not sedated with midazolam but no difference in the duration of hospitalization when compared with patients who were sedated with propofol and dexmedetomidine. Patients sedated with propofol had no difference in ventilation duration compared with patients who were not sedated with propofol and those who were sedated with dexmedetomidine. Additionally, patients who were sedated with propofol had a longer ICU stay than those who were not sedated with propofol and no difference in ICU stay duration compared with those who were sedated with dexmedetomidine. Furthermore, patients sedated with propofol had a longer hospital stay than those not sedated with propofol and no difference in hospitalization duration compared with those sedated with dexmedetomidine. The use of dexmedetomidine was associated with an longer ventilation duration, ICU stay and hospital stay than the use of other sedatives.

Patients sedated with midazolam had a lower rate of being discharged home than patients not sedated with midazolam (Table 2), those sedated with propofol and those sedated with dexmedetomidine. Patients sedated with propofol had no difference in the rate of being discharged home compared with patients who were not sedated with propofol and those who were sedated with dexmedetomidine. Patients sedated with dexmedetomidine also had no difference in the rate of being discharged home compared with patients who were not sedated with dexmedetomidine.

### Multivariable Analysis

The results of the multivariable analysis of five outcomes among patients who were sedated with midazolam, propofol, and dexmedetomidine are shown in Table 3. Patients treated with midazolam had no difference in mortality when compared with patients who were not sedated with midazolam but had a higher mortality rate than patients sedated with propofol and those sedated with dexmedetomidine. Patients sedated with propofol had a lower mortality rate than patients who were not sedated with propofol and, unexpectedly, a higher mortality rate than patients sedated with dexmedetomidine. Patients sedated with dexmedetomidine had a lower mortality rate than patients who were not sedated with dexmedetomidine.

**TABLE 3:** Results of multivariate analysis in patients with or at risk for acute respiratory distress syndrome.

	Comparator	Hospital mortality^a^	Ventilator days^b^	ICU days^b^	Hospital days^b^	Discharge to home^a^
Midazolam	No Midazolam	0.99 (0.89–1.11), 0.90	0.14 (0.03–0.25), <0.01	4.14 (3.76–4.51), <0.001	3.61 (2.89–4.32), <0.001	0.81 (0.73–0.89), <0.001
Midazolam	Propofol	1.43 (1.22–1.66), <0.001	0.35 (0.19–0.50), <0.001	1.01 (0.57–1.45), <0.001	0.99 (0.12–1.87), 0.03	0.72 (0.62–0.83), <0.001
Midazolam	Dex	2.30 (1.89–2.79), <0.001	0.13 (−0.06–0.32), 0.18	2.84 (2.08–3.59), <0.001	1.26 (−0.13–2.66), 0.08	0.60 (0.51–0.70), <0.001
Propofol	No Propofol	0.75 (0.69–0.81), <0.001	−0.19 (−0.26–0.11), <0.001	1.21 (0.94–1.48), <0.001	0.66 (0.15–1.18), <0.01	1.18 (1.11–1.27), <0.001
Propofol	Dex	1.75 (1.40–2.23), <0.001	−0.06 (−0.24–0.12), 0.52	0.79 (0.08–1.50), 0.03	−0.94 (−2.31–0.44), 0.18	0.83 (0.70–0.98), 0.03
Dex	No Dex	0.50 (0.44–0.57), <0.001	0.08 (-0.04–0.19), 0.18	1.53 (1.13–1.93), <0.001	2.27 (1.52–3.03), <0.001	1.19 (1.15–1.40), <0.001

**Abbreviation:** Dex, dexmedetomidine.

^a^ Data are presented as odds ratio (95% confidence interval), *p* value.

^b^ Data are presented as difference of variable value (95% confidence interval), *P* value.

Patients sedated with midazolam had a significantly longer ventilation duration than those who were not sedated with midazolam (Table 3) and those who were sedated with propofol but had no difference in ventilation duration compared with those sedated with dexmedetomidine. Patients sedated with midazolam had a longer ICU stay than those not sedated with midazolam, those sedated with propofol and those sedated with dexmedetomidine. Similarly, patients sedated with midazolam had a longer hospital stay than those not sedated with midazolam and those sedated with propofol but they had no difference in hospitalization duration compared with patients sedated with dexmedetomidine. Patients sedated with propofol had a significantly shorter ventilation duration than those not sedated with propofol but no difference in ventilation duration compared with patients sedated with dexmedetomidine. However, patients sedated with propofol had a longer ICU stay than those not sedated with propofol and those sedated with dexmedetomidine. In addition, patients sedated with propofol had a longer hospital stay than those not sedated with propofol but no difference in hospitalization duration compared with those sedated with dexmedetomidine. Compared with patients not sedated with dexmedetomidine, those sedated with dexmedetomidine did not have a different ventilation duration but had a longer ICU stay and longer hospital stay.

Patients who were sedated with midazolam had a lower likelihood of being discharged home than patients not sedated with midazolam (Table 3), patients sedated with propofol, and patients sedated with dexmedetomidine. Patients sedated with propofol had a higher rate of being discharged home than patients who were not sedated with propofol but a lower rate of being discharged home than patients sedated with dexmedetomidine. The rate of being discharged home was significantly elevated for patients sedated with dexmedetomidine.

### Subgroup Analysis

**FIGURE 1:**
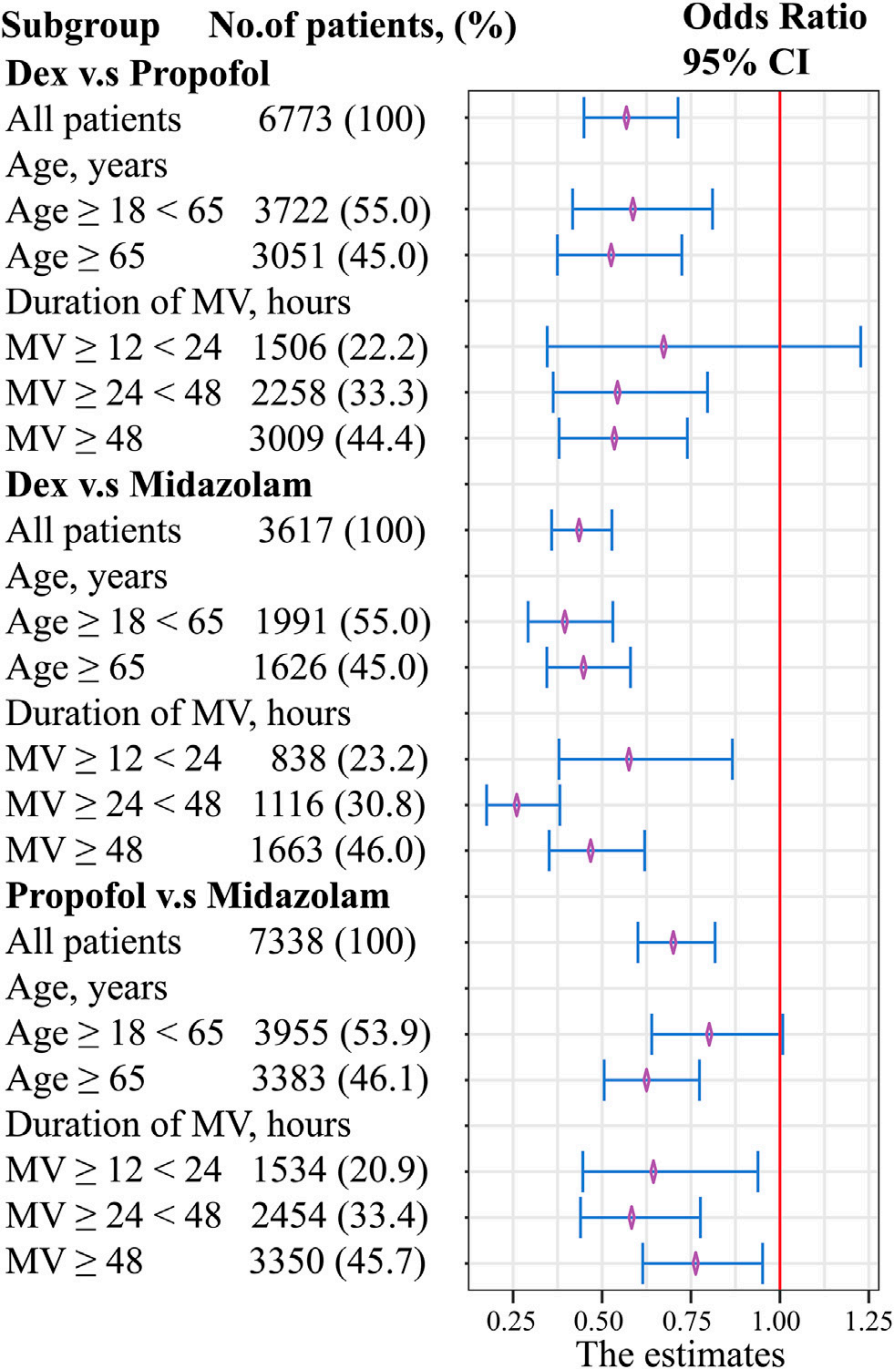
Forest plot evaluating the relationship between age, duration of mechanical ventilation, and sedatives. Abbreviations: MV, mechanical ventilation.

To examine the impact of different sedation agent administration on mortality across a subset of patients, age and duration of mechanical ventilation were explored in the stratified analysis **(** Figure 1).

Dexmedetomidine showed a decrease in mortality with respect to age factor compared with propofol. However, no significant difference was found when duration of MV between 12-24 h (OR, 0.67; 95% CI, 0.34–1.23; *p* = 0.218). No clinically meaningful differences were found with respect to the age and duration of MV factor between dexmedetomidine and midazolam. Propofol showed a decrease in mortality with respect to age factor compared with midazolam. However, no significant difference was found when age factor between 18-65 h (OR, 0.80; 95% CI, 0.64–1.01; *p* = 0.056).

## Discussion

MFAS was a multicenter, observational cohort study performed to provide evidence about the risks associated with sedation with midazolam, propofol, and dexmedetomidine. All pairwise comparisons between the routine sedatives were examined for a set of outcomes. We demonstrated that compared with sedation with dexmedetomidine, sedation without dexmedetomidine and sedation with midazolam were associated with higher in-hospital mortality rates. Likewise, sedation with propofol was associated with a lower mortality rate than sedation without propofol. Additionally, when compared with no sedation, the use of midazolam, propofol or dexmedetomidine was associated with a longer ICU stay and longer hospitalization duration. In addition, there were no significant associations between ventilation duration, length of ICU stay, and length of hospitalization and treatment with propofol or dexmedetomidine. Finally, patients who were sedated with midazolam were significantly less likely to be discharged home than patients not sedated with midazolam and those sedated with propofol or dexmedetomidine.

A previous systematic review demonstrated that sedation with dexmedetomidine was associated with a lower 28 days mortality rate than sedation with other agents in patients with sepsis (Zamani et al., 2016). Similarly, sedation with dexmedetomidine was associated with an 8% reduction in the 28 days mortality rate compared with sedation with midazolam in 201 patients with sepsis undergoing ventilation; unfortunately, the statistical analysis in that study lacked sufficient power to detect differences (Kawazoe et al., 2017). In another recent study, the use of dexmedetomidine for light sedation in patients undergoing mechanical ventilation was associated with a similar 90 days mortality rate as the use of midazolam, propofol or other sedatives (Shehabi et al., 2019). Nevertheless, 64% of patients in the dexmedetomidine group in that study received supplemental propofol to achieve the necessary sedation level after randomization. However, a systematic comparison of the effectiveness and safety of individual sedatives to determine which sedative leads to better prognosis in mechanically ventilated ICU patients has been lacking.

No significant difference was found between dexmedetomidine and propofol when duration of MV between 12-24 h, and between propofol and midazolam when age between 18-65 years. Compared with midazolam use, dexmedetomidine use or propofol use might not be associated with beneficial effect in reducing in-hospital mortality in young patients with mild disease. However, dexmedetomidine showed a decrease in-hospital mortality rates with respect to age or duration of MV factor compared with midazolam. In general, our findings indicate that dexmedetomidine may be superior to midazolam or propofol.

There are multiple mechanisms by which dexmedetomidine may reduce the incidence of lung injury and thus lead to a lower rate of mortality than other sedatives. In patients with sepsis, sedation with dexmedetomidine was associated with lower rates and shorter durations of coma and delirium (Riker et al., 2009; Pandharipande et al., 2010; Skrobik et al., 2018; Wang et al., 2019), both of which are independently associated with an increased mortality rate and prolonged hospitalization duration (Ely et al., 2004; Pisani et al., 2009; Shehabi et al., 2010; Shi et al., 2019). Additionally, respiratory drive affects the pathophysiology and clinical outcome of ARDS (Spinelli et al., 2020). Dexmedetomidine was not found to affect the respiratory rate or gas exchange in ICU patients compared to a placebo (Venn et al., 2000) and did not affect the hypercapnic ventilatory response in healthy volunteers (Hsu et al., 2004); conversely, sedation with midazolam or propofol might suppress the respiratory drive in patients on mechanical ventilation (Migliari et al., 2009; Rozé et al., 2015). Another biological rationale for the potential benefit of dexmedetomidine is based on the experimental evidence of protective effects against neuronal, myocardial, and renal injury (Si et al., 2014; Ren et al., 2016), the reduction in the levels of inflammatory mediators after cardiopulmonary bypass; and the reduced mortality rates observed in animal models (Taniguchi et al., 2004; Ueki et al., 2014). Taken together, these findings provide a rationale for the possible reduction in mortality associated with the use of dexmedetomidine (Shehabi et al., 2012; Shehabi et al., 2013c; Shehabi et al., 2018; Stephens et al., 2018).

In this context, our findings add information regarding the clinical management of patients with ARDS. In total, 41.4% of the patients did not receive common opioids or sedatives in our study. Although the cohort of patients who received midazolam, propofol or dexmedetomidine had more severe disease at baseline, even after adjustment, we found that patients who received a sedative had a longer ICU stay than those who did not receive sedation.

The 2013 PAD guidelines state that the use of benzodiazepine is a risk factor for the development of delirium in adult ICU patients (Barr et al., 2013). Moreover, the 2018 Clinical Practice Guidelines suggest using propofol rather than benzodiazepine for sedation in mechanically ventilated adults after cardiac surgery (Devlin et al., 2018). Likewise, in our study, patients who were sedated with midazolam had similar mortality, longer ventilation, longer LOS of ICU and hospital, and less likely to be discharged home than those sedated with other agents in both the propensity score-matched model and the linear regression.

Our study has four strengths. First, our study systematically evaluated all key variables of sedation in clinical practice. These have not often been assessed together in other studies on sedation. Second, our large sample size provided sufficient statistical power to fit a stable model despite the large number of covariates and thus to detect associations between sedatives and outcomes. Third, this study used data from multiple ICU databases from across a range of hospital and ICU settings. The resulting large-scale, unfiltered population more accurately represented real-world practice than the restricted study populations in the prescribed treatment and follow-up settings in clinical trials. Fourth, dexmedetomidine was used as a matter of course in patients with more severe disease in three cohorts (Supplementary Materials S9–S11). Patients treated with dexmedetomidine had a lower oxygenation index and a higher AaDo2. However, we still demonstrated that the use of dexmedetomidine was associated with a reduced rate of mortality.

Our study had three limitations. First, we only compared the outcomes between different sedatives. There were also some missing data for multiple confounding variables, and some variable could not be effectively merged or compared, such as different drug doses, treatment durations, target sedation levels or daily data on sedation levels. Bias may still exist despite the use of propensity score matching and regression modeling to control for a variety of patient and hospital confounders. Second, the results of the propensity score-matched analysis and linear regression only included a subset of the databases from the United States, and we cannot exclude the possibility that certain subpopulations were not adequately represented in our different cohort groups, and such subpopulations could have markedly different effectiveness profiles. Third, a subgroup analysis was performed on the duration of mechanical ventilation; however, the time-varying nature of the sedatives and the covariates was not included in this study. Thus, our results should be applied cautiously to patients with or at risk for ARDS.

## Conclusion

The use of dexmedetomidine was independently associated with a reduced in-hospital mortality rate in patients with or at risk for ARDS. Sedation with midazolam, propofol or dexmedetomidine was associated with longer ICU stays and hospital stays. Sedation with midazolam was independently associated with a lower likelihood of being discharged home. Therefore, dexmedetomidine may be the best choice for a sedative in these patients. Further studies are needed to evaluate the mechanism underlying these differences and to validate these findings in other cohorts of patients.

## Data Availability

The datasets presented in this study can be found in online repositories. The names of the repository/repositories and accession number(s) can be found in the article/Supplementary Material.
