# Supplementary material for: Comparative Effectiveness of Midazolam, Propofol, and Dexmedetomidine in Patients With or at Risk for Acute Respiratory Distress Syndrome: A Propensity Score-Matched Cohort Study
Source: Front Pharmacol. 2021 Mar 23;12:614465. doi: 10.3389/fphar.2021.614465 (PMC8044880; doi:10.3389/fphar.2021.614465)
Supplement: Supplementary file 1 [file datasheet1.docx]

**Comparative efficacy of fentanyl and morphine in patients with or at risk for acute respiratory distress syndrome: a propensity-matched cohort study**

**SUPPLEMENTAL MATERIAL**

**Supplement file 1.** ICD-9-CM codes to as inclusion criteria for patient analysis

**Supplement file 2.** Amount of missing data for each variable included in the analysis

**Supplement file 3.** Distributions of predictors before and after multiple imputations

**Supplement file 4.** STROBE Statement—Checklist of items that should be included in reports of *cohort studies*

**Supplement file 5.** The flow of participants through the study

**Additional file 6.** Demographics and clinical characteristics of the patients who received midazolam or not before and after propensity score adjustment

**Additional file 7.** Demographics and clinical characteristics of the patients who received propofol or not before and after propensity score adjustment

**Additional file 8.** Demographics and clinical characteristics of the patients who received dexmedetomidine or not before and after propensity score adjustment

**Additional file 9.** Demographics and clinical characteristics of the patients who received midazolam or propofol before and after propensity score adjustment

**Additional file 10.** Demographics and clinical characteristics of the patients who received midazolam or dexmedetomidine before and after propensity score adjustment

**Additional file 11.** Demographics and clinical characteristics of the patients who received propofol or dexmedetomidine before and after propensity score adjustment

This supplementary material has been provided by the authors to give readers additional information about their work.

**Supplement file 1. ICD-9-CM codes to as inclusion criteria for patient analysis**

| Disease | ICD-9-CM codes |
| --- | --- |
| Acute respiratory distress syndrome or Acute hypoxemic respiratory failure | 518.0, 518.5, 518.51, 518.53, 518.81, 518.84, 799.1 |
| Penumonia (bacterial, viral, fungal or opportunistic) | 003.22, 041.3, 073.0, 115.05, 115.95, 480.0, 480.2, 480.3, 480.8, 480.9, 481, 482.0, 482.1, 482.2, 482.30, 482.31, 482.32, 482.39, 482.40, 482.41, 482.42, 482.49, 482.81, 482.82, 482.83, 482.84, 482.89, 482.9, 483.0, 483.1, 483.8, 484.1, 484.3, 484.5, 484.6, 484.7, 484.8, 485, 486, 487.0, 488.01, 488.11, 488.81, 997.31, 997.32, V03.82, V06.6, V12.61 |
| Sepsis | 003.1, 038.0, 038.10, 038.11, 038.12, 038.19, 038.2, 038.3, 038.40, 038.41, 038.42, 038.43, 038.44, 038.49, 038.8, 038.9, 054.5, 567.9, 659.31, 659.33, 670.22, 670.24, 790.7, 995.91, 995.92, 998.02, 995.59 |
| Trauma | 34.04, 780.33, 795.59, 807.4, 958.3, 958.4, 958.7, 958.8, 958.93 |
| Aspiration of gastic contents or Inhalation injury | 504, 507.0, 507.1, 508.2, 770.85, 778.6 |
| Other | 39.61, 54.11, 54.62, 99.04, 99.05, 99.07, 285.1, 305.00, 305.01, 305.02, 305.03, 348.1, 427.5, 458.9, 511.9, 518.7, 570, 577.0, 577.1, 790.3, 861.32, 977.3, 994.1, 994.7, E953.0 |

**Supplement file 2. Amount of missing data for each variable included in the analysis**

|  | Missing data (n) | Missing data (%) |
| --- | --- | --- |
| AaDO2 | 3419 | 19.64 |
| Admitted ICU type | 0 | 0.00 |
| Age | 24 | 0.14 |
| APACHE-III score | 187 | 1.07 |
| Chronic pulmonary disease | 0 | 0.00 |
| Diabetess | 0 | 0.00 |
| Ethnicity | 107 | 0.61 |
| Heart failure | 0 | 0.00 |
| Height | 163 | 0.94 |
| Hypertension | 0 | 0.00 |
| Liver disease | 0 | 0.00 |
| Number of beds | 1327 | 7.62 |
| Oxgenation index | 3211 | 18.44 |
| Provider region | 864 | 4.96 |
| Renal failure | 0 | 0.00 |
| Sex | 10 | 0.06 |
| Teaching | 0 | 0.00 |
| Weight | 122 | 0.70 |
| Abbreviations: AaDO2, Alveolar-arterial Oxygen Difference; APACHE-III score: the acute physiology and chronic health evaluation III score. | | |
|  |  |  |

**Supplement file 3. Distributions of predictors before and after multiple imputations**


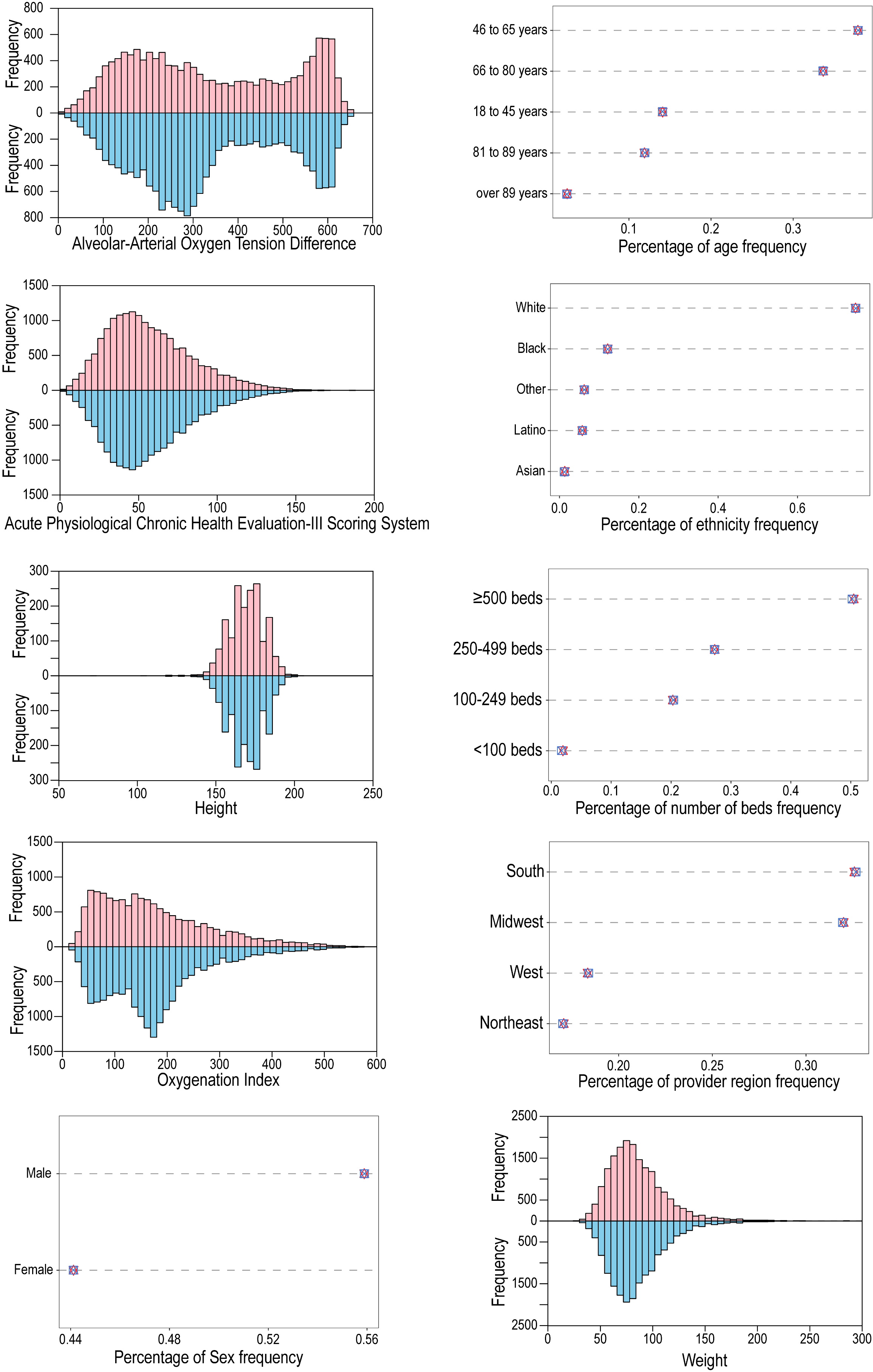


Legend: The distributions for each variable that were included in the imputation models. Before imputations were shown in red and after multiple imputations in blue.

**Supplement file 4. STROBE Statement—Checklist of items that should be included in reports of *cohort studies***

|  | Item No | Recommendation | Section |
| --- | --- | --- | --- |
| **Title and abstract** | 1 | (*a*) Indicate the study’s design with a commonly used term in the title or the abstract | Title |
|  |  | (*b*) Provide in the abstract an informative and balanced summary of what was done and what was found | Title/abstract |
| Introduction | | |  |
| Background/rationale | 2 | Explain the scientific background and rationale for the investigation being reported | Introduction Par.1-2 |
| Objectives | 3 | State specific objectives, including any prespecified hypotheses | Introduction Par.3 |
| Methods | | |  |
| Study design | 4 | Present key elements of study design early in the paper | Methods Par.1 |
| Setting | 5 | Describe the setting, locations, and relevant dates, including periods of recruitment, exposure, follow-up, and data collection | Methods Par.1 |
| Participants | 6 | (*a*) Give the eligibility criteria, and the sources and methods of selection of participants. Describe methods of follow-up | Methods Par.2-3 |
|  |  | (*b*) For matched studies, give matching criteria and number of exposed and unexposed | Methods Par.4 |
| Variables | 7 | Clearly define all outcomes, exposures, predictors, potential confounders, and effect modifiers. Give diagnostic criteria, if applicable | Methods Par.5-6 |
| Data sources/ measurement | 8* | For each variable of interest, give sources of data and details of methods of assessment (measurement). Describe comparability of assessment methods if there is more than one group | Methods Par.4-8 |
| Bias | 9 | Describe any efforts to address potential sources of bias | Methods Par.6-7 |
| Study size | 10 | Explain how the study size was arrived at | Methods Par.1 |
| Quantitative variables | 11 | Explain how quantitative variables were handled in the analyses. If applicable, describe which groupings were chosen and why | Methods Par.4-6 |
| Statistical methods | 12 | (*a*) Describe all statistical methods, including those used to control for confounding | Methods Par.6-8 |
|  |  | (*b*) Describe any methods used to examine subgroups and interactions | Methods Par.6-7 |
|  |  | (*c*) Explain how missing data were addressed | Methods Par.8 |
|  |  | (*d*) If applicable, explain how loss to follow-up was addressed | Methods Par.8 |
|  |  | (*e*) Describe any sensitivity analyses | NA |
| Results | | |  |
| Participants | 13* | (a) Report numbers of individuals at each stage of study—eg numbers potentially eligible, examined for eligibility, confirmed eligible, included in the study, completing follow-up, and analysed | Results Par.1 |
|  |  | (b) Give reasons for non-participation at each stage | Results Par.1 |
|  |  | (c) Consider use of a flow diagram | Supplement file 5 |
| Descriptive data | 14* | (a) Give characteristics of study participants (eg demographic, clinical, social) and information on exposures and potential confounders | Results Par.1-2, Supplement file 6-11 |
|  |  | (b) Indicate number of participants with missing data for each variable of interest | Supplement file 2-3 |
|  |  | (c) Summarise follow-up time (eg, average and total amount) | Methods Par.1-2 |
| Outcome data | 15* | Report numbers of outcome events or summary measures over time | Methods Par.1 |
| Main results | 16 | (*a*) Give unadjusted estimates and, if applicable, confounder-adjusted estimates and their precision (eg, 95% confidence interval). Make clear which confounders were adjusted for and why they were included | Results Par.3-8 |
|  |  | (*b*) Report category boundaries when continuous variables were categorized | Methods Par.3-8 |
|  |  | (*c*) If relevant, consider translating estimates of relative risk into absolute risk for a meaningful time period | Table 2, Table 3 |
| Other analyses | 17 | Report other analyses done—eg analyses of subgroups and interactions, and sensitivity analyses | Methods Par.3-8 |
| Discussion | | |  |
| Key results | 18 | Summarise key results with reference to study objectives | Discussion Par.1 |
| Limitations | 19 | Discuss limitations of the study, taking into account sources of potential bias or imprecision. Discuss both direction and magnitude of any potential bias | Discussion Par.7 |
| Interpretation | 20 | Give a cautious overall interpretation of results considering objectives, limitations, multiplicity of analyses, results from similar studies, and other relevant evidence | Discussion Par.2-7 |
| Generalisability | 21 | Discuss the generalisability (external validity) of the study results | Discussion Par.7 |
| Other information | | |  |
| Funding | 22 | Give the source of funding and the role of the funders for the present study and, if applicable, for the original study on which the present article is based | Title Page |

*Give information separately for exposed and unexposed groups.

**Note:** An Explanation and Elaboration article discusses each checklist item and gives methodological background and published examples of transparent reporting. The STROBE checklist is best used in conjunction with this article (freely available on the Web sites of PLoS Medicine at http://www.plosmedicine.org/, Annals of Internal Medicine at http://www.annals.org/, and Epidemiology at http://www.epidem.com/). Information on the STROBE Initiative is available at http://www.strobe-statement.org.

**Supplement file 5. The flow of participants through the study**


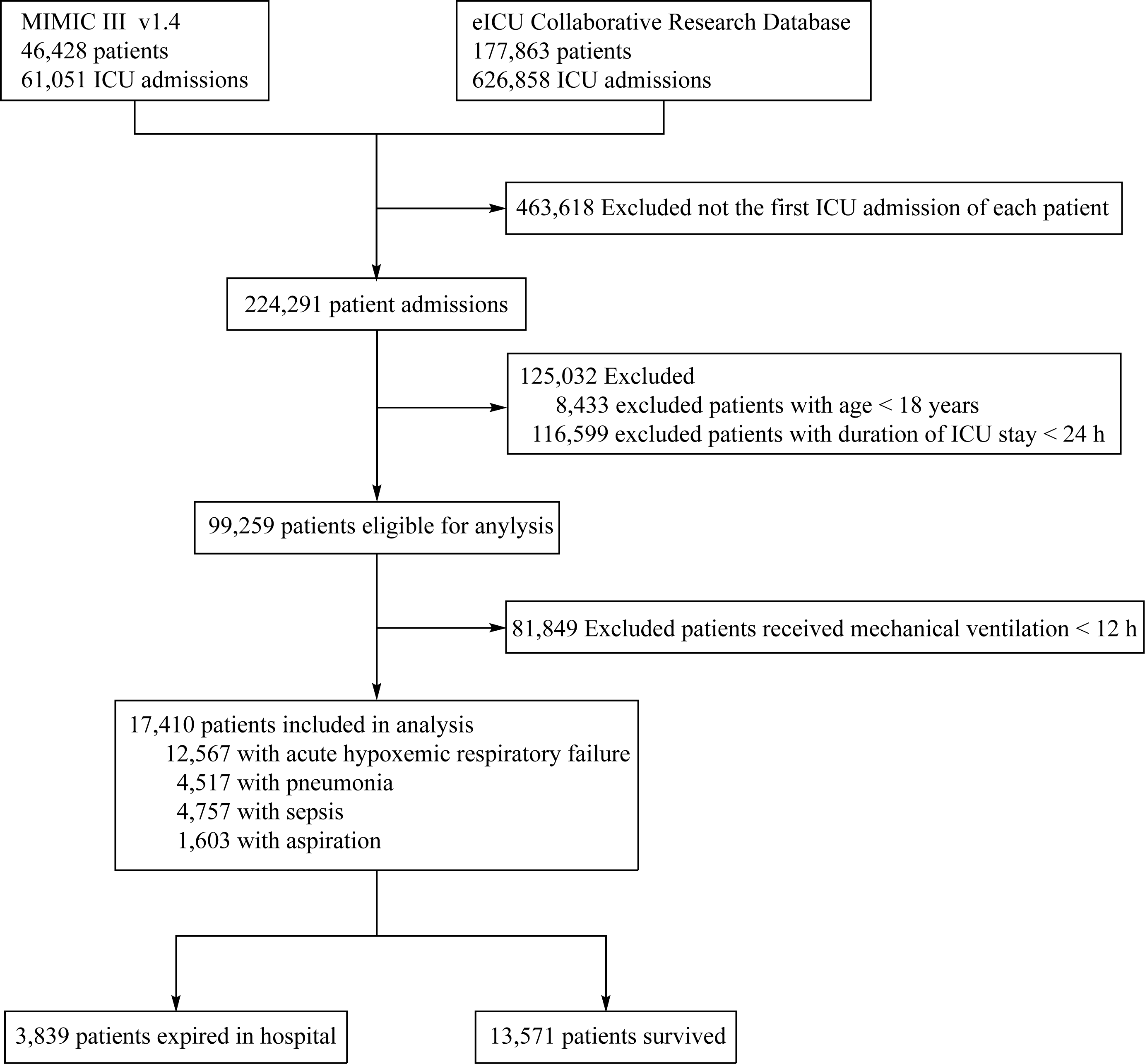


**Additional file 6. Demographics and clinical characteristics of the patients who received midazolam or not before and after propensity score adjustment**

| **Clinical Variable*** | **Baseline covariates of patient  before Propensity Matching** | | | |  | **Baseline covariates of patient  after Propensity Matching** | | | |
| --- | --- | --- | --- | --- | --- | --- | --- | --- | --- |
|  | **Midazolam (N=2,719)** | **No Midazolam (N=14,691)** | **SD^#^** | ***P* value** |  | **Midazolam (N=1,921)** | **No Midazolam (N=1,921)** | **SD** | ***P* value** |
| **Age, No. (%)** |  |  |  |  |  |  |  |  |  |
| 18 to 45 years | 504 (18.5) | 1948 (13.3) | 0.233 | <0.001 |  | 333 (17.3) | 322 (16.8) | 0.026 | 0.960 |
| 46 to 65 years | 1146 (42.1) | 5455 (37.1) |  |  |  | 800 (41.6) | 788 (41) |  |  |
| 66 to 80 years | 797 (29.3) | 5062 (34.5) |  |  |  | 579 (30.1) | 594 (30.9) |  |  |
| 81 to 89 years | 229 (8.4) | 1838 (12.5) |  |  |  | 175 (9.1) | 181 (9.4) |  |  |
| over 89 years | 43 (1.6) | 388 (2.6) |  |  |  | 34 (1.8) | 36 (1.9) |  |  |
| **Female, No. (%)** | 1123 (41.3) | 6556 (44.6) | 0.067 | 0.001 |  | 1129 (58.8) | 1107 (57.6) | 0.023 | 0.492 |
| **Ethnicity, No. (%)** |  |  |  |  |  |  |  |  |  |
| White | 2101 (77.3) | 10891 (74.1) | 0.132 | <0.001 |  | 1442 (75.1) | 1440 (75) | 0.052 | 0.634 |
| Black | 240 (8.8) | 1872 (12.7) |  |  |  | 197 (10.3) | 209 (10.9) |  |  |
| Latino | 153 (5.6) | 844 (5.7) |  |  |  | 133 (6.9) | 114 (5.9) |  |  |
| Asian | 32 (1.2) | 197 (1.3) |  |  |  | 26 (1.4) | 23 (1.2) |  |  |
| Other | 193 (7.1) | 887 (6) |  |  |  | 123 (6.4) | 135 (7) |  |  |
| **Weight, kg** | 83.1 (68.0-100.0) | 80.0 (66.4-98.0) | 0.103 | <0.001 |  | 83.0 (67.2-100.0) | 80.7 (67.3-98.8) | 0.041 | 0.207 |
| **Height, cm** | 170.2 (162.6-177.8) | 170 (162.5-177.8) | 0.104 | <0.001 |  | 170.2 (162.6-177.8) | 170.2 (162.6-177.8) | 0.013 | 0.688 |
| **APACHE-III score** | 61 (42-83) | 52 (37-71.9) | 0.307 | <0.001 |  | 58 (41-80) | 59 (42-81) | 0.014 | 0.668 |
| **Oxgenation index, mmHg** | 129.0 (68.0-190.0) | 170.7 (114.3-222.5) | 0.407 | <0.001 |  | 142.0 (78.0-197.4) | 142.9 (81.0-194.0) | 0.002 | 0.956 |
| **AaDo2, mmHg** | 361.6 (241.0-562.6) | 287.5 (204.3-447.8) | 0.381 | <0.001 |  | 344.8 (231-555.5) | 344 (233.7-535.1) | 0.009 | 0.778 |
| **Patient characteristics at discharge, No. (%)** |  |  |  |  |  |  |  |  |  |
| ARDS | 2226 (81.9) | 10341 (70.4) | 0.272 | <0.001 |  | 1481 (77.1) | 1473 (76.7) | 0.010 | 0.789 |
| Pneumonia | 1043 (38.4) | 3474 (23.6) | 0.322 | <0.001 |  | 591 (30.8) | 592 (30.8) | 0.001 | 1.000 |
| Sepsis | 941 (34.6) | 3816 (26) | 0.189 | <0.001 |  | 572 (29.8) | 554 (28.8) | 0.021 | 0.547 |
| Aspiration | 396 (14.6) | 1207 (8.2) | 0.201 | <0.001 |  | 220 (11.5) | 211 (11) | 0.015 | 0.683 |
| Heart failure | 220 (8.1) | 794 (5.4) | 0.107 | <0.001 |  | 121 (6.3) | 122 (6.4) | 0.002 | 1.000 |
| Chronic pulmonary disease | 445 (16.4) | 2002 (13.6) | 0.077 | <0.001 |  | 273 (14.2) | 261 (13.6) | 0.018 | 0.608 |
| Liver disease | 106 (3.9) | 269 (1.8) | 0.124 | <0.001 |  | 44 (2.3) | 44 (2.3) | <0.001 | 1.000 |
| Renal failure | 315 (11.6) | 1742 (11.9) | 0.008 | 0.710 |  | 199 (10.4) | 205 (10.7) | 0.010 | 0.793 |
| Hypertension | 535 (19.7) | 2355 (16) | 0.095 | <0.001 |  | 315 (16.4) | 306 (15.9) | 0.013 | 0.726 |
| Diabetes | 174 (6.4) | 402 (2.7) | 0.176 | <0.001 |  | 90 (4.7) | 97 (5) | 0.017 | 0.653 |
| **Hospital characteristics** |  |  |  |  |  |  |  |  |  |
| **ICU type, No. (%)** |  |  |  |  |  |  |  |  |  |
| SICU | 360 (13.2) | 1339 (9.1) | 0.161 | <0.001 |  | 267 (13.9) | 255 (13.3) | 0.022 | 0.926 |
| CCU | 503 (18.5) | 2892 (19.7) |  |  |  | 324 (16.9) | 335 (17.4) |  |  |
| NICU | 107 (3.9) | 901 (6.1) |  |  |  | 94 (4.9) | 93 (4.8) |  |  |
| Others | 1749 (64.3) | 9559 (65.1) |  |  |  | 1236 (64.3) | 1238 (64.4) |  |  |
| **Number of beds, No. (%)** |  |  |  |  |  |  |  |  |  |
| <100 | 19 (0.7) | 320 (2.2) | 0.331 | <0.001 |  | 14 (0.7) | 8 (0.4) | 0.052 | 0.453 |
| 100-249 | 470 (17.3) | 3063 (20.8) |  |  |  | 404 (21) | 425 (22.1) |  |  |
| 250-499 | 509 (18.7) | 4240 (28.9) |  |  |  | 463 (24.1) | 474 (24.7) |  |  |
| ≥500 | 1721 (63.3) | 7068 (48.1) |  |  |  | 1040 (54.1) | 1014 (52.8) |  |  |
| **Teaching, No. (%)** | 1334 (49.1) | 4222 (28.7) | 0.426 | <0.001 |  | 657 (34.2) | 643 (33.5) | 0.015 | 0.658 |
| **Provider region, No. (%)** |  |  |  |  |  |  |  |  |  |
| Midwest | 587 (21.6) | 4914 (33.4) | 0.774 | <0.001 |  | 553 (28.8) | 575 (29.9) | 0.030 | 0.837 |
| Northeast | 1168 (43) | 1676 (11.4) |  |  |  | 475 (24.7) | 457 (23.8) |  |  |
| South | 705 (25.9) | 5116 (34.8) |  |  |  | 637 (33.2) | 639 (33.3) |  |  |
| West | 259 (9.5) | 2985 (20.3) |  |  |  | 256 (13.3) | 250 (13) |  |  |
| **Using first-line sedation or opioids drugs, No. (%)** |  |  |  |  |  |  |  |  |  |
| Propofol | 1470 (54.1) | 6089 (41.4) | 0.255 | <0.001 |  | 1230 (64) | 1225 (63.8) | 0.005 | 0.893 |
| Dexmedetomidine | 668 (24.6) | 1566 (10.7) | 0.371 | <0.001 |  | 529 (27.5) | 519 (27) | 0.012 | 0.744 |
| Morphine | 261 (9.6) | 340 (2.3) | 0.311 | <0.001 |  | 133 (6.9) | 123 (6.4) | 0.021 | 0.671 |
| Fentanyl | 2102 (77.3) | 3413 (23.2) | 1.286 | <0.001 |  | 1360 (70.8) | 1347 (70.1) | 0.015 | 0.560 |
| Not using the four drugs | 775 (28.5) | 5325 (36.2) | 1.387 | <0.001 |  | 596 (31) | 650 (33.8) | 0.032 | 0.067 |

Abbreviations: AaDO2, alveolar-arterial oxygen difference; APACHE-III score, the acute physiology and chronic health evaluation III score; CCU, cardiac care unit; Dex, dexmedetomidine; NICU, neurological intensive care unit; SICU, surgical intensive care unit.

* Data shown as mean ± standard deviation, number (percent), or median (interquartile range) as appropriate.

# SD = standardized difference (SD ≥ 0.1 represent significant differences in covariables between groups).

**Additional file 7. Demographics and clinical characteristics of the patients who received Propofol or not before and after propensity score adjustment**

| **Clinical Variable*** | **Baseline covariates of patient  before Propensity Matching** | | | |  | **Baseline covariates of patient  after Propensity Matching** | | | |
| --- | --- | --- | --- | --- | --- | --- | --- | --- | --- |
|  | **Propofol (N=7,559)** | **No Propofol (N=9,851)** | **SD^#^** | ***P* value** |  | **Propofol (N=2,256)** | **No Propofol (N=2,256)** | **SD** | ***P* value** |
| **Age, No. (%)** |  |  |  |  |  |  |  |  |  |
| 18 to 45 years | 1225 (16.2) | 1227 (12.5) | 0.166 | <0.001 |  | 283 (12.5) | 281 (12.5) | 0.012 | 0.997 |
| 46 to 65 years | 3002 (39.7) | 3599 (36.5) |  |  |  | 862 (38.2) | 853 (37.8) |  |  |
| 66 to 80 years | 2411 (31.9) | 3448 (35) |  |  |  | 786 (34.8) | 791 (35.1) |  |  |
| 81 to 89 years | 787 (10.4) | 1280 (13) |  |  |  | 277 (12.3) | 284 (12.6) |  |  |
| over 89 years | 134 (1.8) | 297 (3) |  |  |  | 48 (2.1) | 47 (2.1) |  |  |
| **Female, No. (%)** | 4298 (56.9) | 5433 (55.2) | 0.034 | 0.025 |  | 1252 (55.5) | 1256 (55.7) | 0.004 | 0.928 |
| **Ethnicity, No. (%)** |  |  |  |  |  |  |  |  |  |
| White | 5699 (75.4) | 7293 (74) | 0.219 | <0.001 |  | 1720 (76.2) | 1731 (76.7) | 0.019 | 0.981 |
| Black | 682 (9) | 1430 (14.5) |  |  |  | 225 (10) | 218 (9.7) |  |  |
| Latino | 578 (7.6) | 419 (4.3) |  |  |  | 123 (5.5) | 124 (5.5) |  |  |
| Asian | 88 (1.2) | 141 (1.4) |  |  |  | 32 (1.4) | 28 (1.2) |  |  |
| Other | 512 (6.8) | 568 (5.8) |  |  |  | 156 (6.9) | 155 (6.9) |  |  |
| **Weight, kg** | 81.6 (68-99.6) | 79.8 (65.7-97.8) | 0.056 | <0.001 |  | 80 (66.8-97.8) | 80.7 (65.4-97) | 0.009 | 0.763 |
| **Height, cm** | 170.2 (162.6-177.8) | 170 (162.5-177.8) | 0.072 | <0.001 |  | 170 (162.6-177.8) | 170.1 (162.6-177.8) | 0.009 | 0.763 |
| **APACHE-III score** | 54 (38-74) | 53 (37-73) | 0.033 | 0.032 |  | 60 (44-80.2) | 60 (42-80) | 0.02 | 0.501 |
| **Oxgenation index, mmHg** | 162.4 (97.9-217.5) | 169 (109-218) | 0.048 | 0.001 |  | 148.1 (82.1-208.6) | 155.3 (87.9-212.5) | 0.027 | 0.356 |
| **AaDo2, mmHg** | 305 (213.6-489.1) | 286.6 (205.5-453.2) | 0.100 | <0.001 |  | 328.7 (221.8-529.6) | 315.1 (213.9-530) | 0.028 | 0.340 |
| **Patient characteristics at discharge, No. (%)** |  |  |  |  |  |  |  |  |  |
| ARDS | 5769 (76.3) | 6798 (69) | 0.165 | <0.001 |  | 1646 (73) | 1666 (73.8) | 0.02 | 0.522 |
| Pneumonia | 1894 (25.1) | 2623 (26.6) | 0.036 | 0.02 |  | 683 (30.3) | 692 (30.7) | 0.009 | 0.796 |
| Sepsis | 1965 (26) | 2792 (28.3) | 0.053 | 0.001 |  | 683 (30.3) | 675 (29.9) | 0.008 | 0.820 |
| Aspiration | 817 (10.8) | 786 (8) | 0.097 | <0.001 |  | 224 (9.9) | 249 (11) | 0.036 | 0.243 |
| Heart failure | 504 (6.7) | 510 (5.2) | 0.063 | <0.001 |  | 128 (5.7) | 143 (6.3) | 0.028 | 0.380 |
| Chronic pulmonary disease | 1051 (13.9) | 1396 (14.2) | 0.008 | 0.631 |  | 334 (14.8) | 339 (15) | 0.006 | 0.867 |
| Liver disease | 150 (2) | 225 (2.3) | 0.021 | 0.195 |  | 59 (2.6) | 63 (2.8) | 0.011 | 0.783 |
| Renal failure | 890 (11.8) | 1167 (11.8) | 0.002 | 0.902 |  | 256 (11.3) | 257 (11.4) | 0.001 | 1.000 |
| Hypertension | 1441 (19.1) | 1449 (14.7) | 0.116 | <0.001 |  | 350 (15.5) | 367 (16.3) | 0.021 | 0.515 |
| Diabetes | 295 (3.9) | 281 (2.9) | 0.058 | <0.001 |  | 113 (5) | 113 (5) | <0.001 | 1.000 |
| **Hospital characteristics** |  |  |  |  |  |  |  |  |  |
| **ICU type, No. (%)** |  |  |  |  |  |  |  |  |  |
| SICU | 706 (9.3) | 993 (10.1) | 0.092 | <0.001 |  | 311 (13.8) | 319 (14.1) | 0.036 | 0.690 |
| CCU | 1405 (18.6) | 1990 (20.2) |  |  |  | 409 (18.1) | 436 (19.3) |  |  |
| NICU | 371 (4.9) | 637 (6.5) |  |  |  | 61 (2.7) | 63 (2.8) |  |  |
| Others | 5077 (67.2) | 6231 (63.3) |  |  |  | 1475 (65.4) | 1438 (63.7) |  |  |
| **Number of beds, No. (%)** |  |  |  |  |  |  |  |  |  |
| <100 | 66 (0.9) | 273 (2.8) | 0.290 | <0.001 |  | 16 (0.7) | 20 (0.9) | 0.021 | 0.918 |
| 100-249 | 1628 (21.5) | 1905 (19.3) |  |  |  | 380 (16.8) | 385 (17.1) |  |  |
| 250-499 | 2501 (33.1) | 2248 (22.8) |  |  |  | 618 (27.4) | 613 (27.2) |  |  |
| ≥500 | 3364 (44.5) | 5425 (55.1) |  |  |  | 1242 (55.1) | 1238 (54.9) |  |  |
| **Teaching, No. (%)** | 1772 (23.4) | 3784 (38.4) | 0.328 | <0.001 |  | 766 (34) | 770 (34.1) | 0.004 | 0.925 |
| **Provider region, No. (%)** |  |  |  |  |  |  |  |  |  |
| Midwest | 2034 (26.9) | 3467 (35.2) | 0.372 | <0.001 |  | 693 (30.7) | 663 (29.4) | 0.044 | 0.543 |
| Northeast | 1199 (15.9) | 1645 (16.7) |  |  |  | 545 (24.2) | 581 (25.8) |  |  |
| South | 2309 (30.5) | 3512 (35.7) |  |  |  | 580 (25.7) | 589 (26.1) |  |  |
| West | 2017 (26.7) | 1227 (12.5) |  |  |  | 438 (19.4) | 423 (18.8) |  |  |
| **Using first-line sedation or opioids drugs, No. (%)** |  |  |  |  |  |  |  |  |  |
| Midazolam | 1470 (19.4) | 1249 (12.7) | 0.185 | <0.001 |  | 860 (38.1) | 915 (40.6) | 0.05 | 0.100 |
| Dexmedetomidine | 1510 (20) | 724 (7.3) | 0.374 | <0.001 |  | 672 (29.8) | 638 (28.3) | 0.033 | 0.279 |
| Morphine | 393 (5.2) | 208 (2.1) | 0.165 | <0.001 |  | 164 (7.3) | 181 (8) | 0.028 | 0.064 |
| Fentanyl | 3606 (47.7) | 1909 (19.4) | 0.629 | <0.001 |  | 1609 (71.3) | 1551 (68.8) | 0.056 | 0.370 |
| Not using the four drugs | 2834 (37.5) | 3266 (33.2) | 2.332 | <0.001 |  | 706 (31.3) | 689 (30.5) | <0.001 | 0.606 |

Abbreviations: AaDO2, alveolar-arterial oxygen difference; APACHE-III score, the acute physiology and chronic health evaluation III score; CCU, cardiac care unit; Dex, dexmedetomidine; NICU, neurological intensive care unit; SICU, surgical intensive care unit.

* Data shown as mean ± standard deviation, number (percent), or median (interquartile range) as appropriate.

# SD = standardized difference (SD ≥ 0.1 represent significant differences in covariables between groups).

**Additional file 8. Demographics and clinical characteristics of the patients who received dexmedetomidine or not before and after propensity score adjustment**

| **Clinical Variable*** | **Baseline covariates of patient  before Propensity Matching** | | | |  | **Baseline covariates of patient  after Propensity Matching** | | | |
| --- | --- | --- | --- | --- | --- | --- | --- | --- | --- |
|  | **Dex (N=2,234)** | **No Dex (N=15,176)** | **SD^#^** | ***P* value** |  | **Dex (N=2,133)** | **No Dex (N=2,133)** | **SD** | ***P* value** |
| **Age, No. (%)** |  |  |  |  |  |  |  |  |  |
| 18 to 45 years | 367 (16.4) | 2085 (13.7) | 0.149 | <0.001 |  | 347 (16.3) | 361 (16.9) | 0.029 | 0.924 |
| 46 to 65 years | 892 (39.9) | 5709 (37.6) |  |  |  | 849 (39.8) | 831 (39) |  |  |
| 66 to 80 years | 736 (32.9) | 5123 (33.8) |  |  |  | 702 (32.9) | 696 (32.6) |  |  |
| 81 to 89 years | 208 (9.3) | 1859 (12.2) |  |  |  | 206 (9.7) | 218 (10.2) |  |  |
| over 89 years | 31 (1.4) | 400 (2.6) |  |  |  | 29 (1.4) | 27 (1.3) |  |  |
| **Female, No. (%)** | 1347 (60.3) | 8384 (55.2) | 0.102 | <0.001 |  | 1281 (60.1) | 1281 (60.1) | <0.001 | 1.000 |
| **Ethnicity, No. (%)** |  |  |  |  |  |  |  |  |  |
| White | 1629 (72.9) | 11363 (74.9) | 0.261 | <0.001 |  | 1564 (73.3) | 1572 (73.7) | 0.034 | 0.867 |
| Black | 192 (8.6) | 1920 (12.7) |  |  |  | 190 (8.9) | 182 (8.5) |  |  |
| Latino | 246 (11) | 751 (4.9) |  |  |  | 216 (10.1) | 216 (10.1) |  |  |
| Asian | 16 (0.7) | 213 (1.4) |  |  |  | 16 (0.8) | 22 (1) |  |  |
| Other | 151 (6.8) | 929 (6.1) |  |  |  | 147 (6.9) | 141 (6.6) |  |  |
| **Weight, kg** | 81.6 (68-99) | 80 (66.4-98.6) | 0.042 | 0.063 |  | 81.6 (68-99) | 81.6 (68-99.7) | 0.001 | 0.971 |
| **Height, cm** | 170.2 (162.6-177.8) | 170 (162.6-177.8) | 0.092 | <0.001 |  | 170.2 (162.6-177.8) | 170.2 (162.6-177.8) | 0.015 | 0.614 |
| **APACHE-III score** | 54 (39-73) | 53 (37-73) | 0.011 | 0.640 |  | 54 (39-74) | 54 (38-74) | 0.018 | 0.549 |
| **Oxgenation index, mmHg** | 144 (83-196.6) | 169.4 (108.3-221) | 0.247 | <0.001 |  | 145 (84-197.1) | 146 (85.8-198.3) | 0.014 | 0.636 |
| **AaDo2, mmHg** | 332.4 (216.6-540) | 290 (206.8-456.5) | 0.224 | <0.001 |  | 330.6 (214.5-536.2) | 328.6 (227.5-535.1) | 0.015 | 0.615 |
| **Patient characteristics at discharge, No. (%)** |  |  |  |  |  |  |  |  |  |
| ARDS | 1715 (76.8) | 10852 (71.5) | 0.120 | <0.001 |  | 1627 (76.3) | 1666 (78.1) | 0.044 | 0.166 |
| Pneumonia | 614 (27.5) | 3903 (25.7) | 0.040 | 0.080 |  | 588 (27.6) | 560 (26.3) | 0.030 | 0.351 |
| Sepsis | 530 (23.7) | 4227 (27.9) | 0.094 | <0.001 |  | 515 (24.1) | 497 (23.3) | 0.020 | 0.541 |
| Aspiration | 267 (12) | 1336 (8.8) | 0.103 | <0.001 |  | 251 (11.8) | 250 (11.7) | 0.001 | 1.000 |
| Heart failure | 126 (5.6) | 888 (5.9) | 0.009 | 0.727 |  | 123 (5.8) | 125 (5.9) | 0.004 | 0.948 |
| Chronic pulmonary disease | 287 (12.8) | 2160 (14.2) | 0.041 | 0.084 |  | 284 (13.3) | 281 (13.2) | 0.012 | 0.928 |
| Liver disease | 31 (1.4) | 344 (2.3) | 0.066 | 0.009 |  | 30 (1.4) | 33 (1.5) | 0.022 | 0.800 |
| Renal failure | 184 (8.2) | 1873 (12.3) | 0.135 | <0.001 |  | 180 (8.4) | 167 (7.8) | 0.019 | 0.502 |
| Hypertension | 372 (16.7) | 2518 (16.6) | 0.002 | 0.968 |  | 341 (16) | 326 (15.3) | 0.019 | 0.555 |
| Diabetes | 103 (4.6) | 473 (3.1) | 0.078 | <0.001 |  | 97 (4.5) | 91 (4.3) | 0.014 | 0.709 |
| **Hospital characteristics** |  |  |  |  |  |  |  |  |  |
| **ICU type, No. (%)** |  |  |  |  |  |  |  |  |  |
| SICU | 165 (7.4) | 1534 (10.1) | 0.267 | <0.001 |  | 161 (7.5) | 145 (6.8) | 0.029 | 0.823 |
| CCU | 468 (20.9) | 2927 (19.3) |  |  |  | 431 (20.2) | 436 (20.4) |  |  |
| NICU | 132 (5.9) | 876 (5.8) |  |  |  | 127 (6) | 127 (6) |  |  |
| Others | 1469 (65.8) | 9839 (64.8) |  |  |  | 1414 (66.3) | 1425 (66.8) |  |  |
| **Number of beds, No. (%)** |  |  |  |  |  |  |  |  |  |
| <100 | 3 (0.1) | 336 (2.2) | 0.267 | <0.001 |  | 3 (0.1) | 4 (0.2) | 0.019 | 0.942 |
| 100-249 | 377 (16.9) | 3156 (20.8) |  |  |  | 377 (17.7) | 382 (17.9) |  |  |
| 250-499 | 530 (23.7) | 4219 (27.8) |  |  |  | 529 (24.8) | 515 (24.1) |  |  |
| ≥500 | 1324 (59.3) | 7465 (49.2) |  |  |  | 1224 (57.4) | 1232 (57.8) |  |  |
| **Teaching, No. (%)** | 522 (23.4) | 5034 (33.2) | 0.219 | <0.001 |  | 507 (23.8) | 511 (24) | 0.004 | 0.914 |
| **Provider region, No. (%)** |  |  |  |  |  |  |  |  |  |
| Midwest | 877 (39.3) | 4624 (30.5) | 0.364 | <0.001 |  | 838 (39.3) | 822 (38.5) | 0.027 | 0.857 |
| Northeast | 191 (8.5) | 2653 (17.5) |  |  |  | 191 (9) | 181 (8.5) |  |  |
| South | 886 (39.7) | 4935 (32.5) |  |  |  | 824 (38.6) | 839 (39.3) |  |  |
| West | 280 (12.5) | 2964 (19.5) |  |  |  | 280 (13.1) | 291 (13.6) |  |  |
| **Using first-line sedation or opioids drugs, No. (%)** |  |  |  |  |  |  |  |  |  |
| Midazolam | 668 (29.9) | 2051 (13.5) | 0.406 | <0.001 |  | 640 (30) | 669 (31.4) | 0.029 | 0.353 |
| Propofol | 1510 (67.6) | 6049 (39.9) | 0.579 | <0.001 |  | 1457 (68.3) | 1505 (70.6) | 0.049 | 0.118 |
| Morphine | 101 (4.5) | 500 (3.3) | 0.063 | <0.001 |  | 101 (4.7) | 104 (4.9) | 0.007 | 0.311 |
| Fentanyl | 1173 (52.5) | 4342 (28.6) | 0.502 | 0.004 |  | 1135 (53.2) | 1169 (54.8) | 0.032 | 0.886 |
| Not using the four drugs | 876 (39.2) | 5224 (34.4) | 1.344 | <0.001 |  | 816 (38.3) | 776 (36.4) | <0.001 | 0.217 |

Abbreviations: AaDO2, alveolar-arterial oxygen difference; APACHE-III score, the acute physiology and chronic health evaluation III score; CCU, cardiac care unit; Dex, dexmedetomidine; NICU, neurological intensive care unit; SICU, surgical intensive care unit.

* Data shown as mean ± standard deviation, number (percent), or median (interquartile range) as appropriate.

# SD = standardized difference (SD ≥ 0.1 represent significant differences in covariables between groups).

**Additional file 9. Demographics and clinical characteristics of the patients who received midazolam or propofol before and after propensity score adjustment**

| **Clinical Variable*** | **Baseline covariates of patient  before Propensity Matching** | | | |  | **Baseline covariates of patient  after Propensity Matching** | | |  |
| --- | --- | --- | --- | --- | --- | --- | --- | --- | --- |
|  | **Midazolam (N=1,249)** | **Propofol (N=6,089)** | **SD^#^** | ***P* value** |  | **Midazolam (N=853)** | **Propofol (N=853)** | **SD** | ***P* value** |
| **Age, No. (%)** |  |  |  |  |  |  |  |  |  |
| 18 to 45 years | 894 (14.7) | 173 (13.9) | 0.055 | 0.537 |  | 143 (16.8) | 139 (16.3) | 0.037 | 0.966 |
| 46 to 65 years | 2372 (39) | 516 (41.3) |  |  |  | 337 (39.5) | 341 (40) |  |  |
| 66 to 80 years | 2024 (33.2) | 410 (32.8) |  |  |  | 275 (32.2) | 270 (31.7) |  |  |
| 81 to 89 years | 689 (11.3) | 131 (10.5) |  |  |  | 85 (10) | 92 (10.8) |  |  |
| over 89 years | 110 (1.8) | 19 (1.5) |  |  |  | 13 (1.5) | 11 (1.3) |  |  |
| **Female, No. (%)** | 2698 (44.3) | 560 (44.8) | 0.011 | 0.757 |  | 380 (44.5) | 385 (45.1) | 0.012 | 0.846 |
| **Ethnicity, No. (%)** |  |  |  |  |  |  |  |  |  |
| White | 4583 (75.3) | 985 (78.9) | 0.139 | 0.002 |  | 650 (76.2) | 652 (76.4) | 0.068 | 0.741 |
| Black | 554 (9.1) | 112 (9.0) |  |  |  | 92 (10.8) | 96 (11.3) |  |  |
| Latino | 483 (7.9) | 58 (4.6) |  |  |  | 41 (4.8) | 48 (5.6) |  |  |
| Asian | 72 (1.2) | 16 (1.3) |  |  |  | 12 (1.4) | 10 (1.2) |  |  |
| Other | 397 (6.5) | 78 (6.2) |  |  |  | 58 (6.8) | 47 (5.5) |  |  |
| **Weight, kg** | 81 (67.6-98.8) | 82 (65-99.8) | 0.024 | 0.431 |  | 80 (67.8-99.7) | 82.5 (66-99.8) | 0.012 | 0.804 |
| **Height, cm** | 170.1 (162.6-177.8) | 170.2 (162.6-177.8) | 0.006 | 0.835 |  | 170.2 (162.6-177.8) | 170.2 (162.6-177.8) | 0.021 | 0.670 |
| **APACHE-III score** | 53 (38-72) | 64 (44-87) | 0.380 | <0.001 |  | 59 (42-82) | 63 (43-84) | 0.103 | 0.034 |
| **Oxgenation index, mmHg** | 170 (113.8-225.0) | 146.4 (75.6-196.8) | 0.291 | <0.001 |  | 151.1 (87.0-204.2) | 152.7 (84.0-200.0) | 0.021 | 0.665 |
| **AaDo2, mmHg** | 293.6 (206.5-455) | 324.9 (229.8-546.8) | 0.243 | <0.001 |  | 318.8 (223.2-496.3) | 317.9 (229.8-534.8) | 0.053 | 0.275 |
| **Patient characteristics at discharge, No. (%)** |  |  |  |  |  |  |  |  |  |
| ARDS | 4553 (74.8) | 1010 (80.9) | 0.147 | <0.001 |  | 640 (75.0) | 640 (75.0) | <0.001 | 1.000 |
| Pneumonia | 1336 (21.9) | 485 (38.8) | 0.374 | <0.001 |  | 232 (27.2) | 251 (29.4) | 0.049 | 0.333 |
| Sepsis | 1469 (24.1) | 445 (35.6) | 0.253 | <0.001 |  | 247 (29) | 262 (30.7) | 0.038 | 0.459 |
| Aspiration | 587 (9.6) | 166 (13.3) | 0.115 | <0.001 |  | 91 (10.7) | 87 (10.2) | 0.015 | 0.812 |
| Heart failure | 399 (6.6) | 115 (9.2) | 0.099 | 0.001 |  | 54 (6.3) | 56 (6.6) | 0.010 | 0.921 |
| Chronic pulmonary disease | 835 (13.7) | 229 (18.3) | 0.126 | <0.001 |  | 137 (16.1) | 122 (14.3) | 0.049 | 0.345 |
| Liver disease | 112 (1.8) | 68 (5.4) | 0.193 | <0.001 |  | 29 (3.4) | 28 (3.3) | 0.007 | 1.000 |
| Renal failure | 742 (12.2) | 167 (13.4) | 0.035 | 0.267 |  | 104 (12.2) | 105 (12.3) | 0.004 | 1.000 |
| Hypertension | 1135 (18.6) | 229 (18.3) | 0.008 | 0.831 |  | 124 (14.5) | 121 (14.2) | 0.010 | 0.890 |
| Diabetes | 201 (3.3) | 80 (6.4) | 0.145 | <0.001 |  | 36 (4.2) | 42 (4.9) | 0.034 | 0.562 |
| **Hospital characteristics** |  |  |  |  |  |  |  |  |  |
| **ICU type, No. (%)** |  |  |  |  |  |  |  |  |  |
| SICU | 492 (8.1) | 146 (11.7) | 0.159 | <0.001 |  | 124 (14.5) | 133 (15.6) | 0.105 | 0.199 |
| CCU | 1163 (19.1) | 261 (20.9) |  |  |  | 168 (19.7) | 148 (17.4) |  |  |
| NICU | 303 (5.0) | 39 (3.1) |  |  |  | 25 (2.9) | 39 (4.6) |  |  |
| Others | 4131 (67.8) | 803 (64.3) |  |  |  | 536 (62.8) | 533 (62.5) |  |  |
| **Number of beds, No. (%)** |  |  |  |  |  |  |  |  |  |
| <100 | 61 (1.0) | 14 (1.1) | 0.608 | <0.001 |  | 5 (0.6) | 4 (0.5) | 0.063 | 0.634 |
| 100-249 | 1371 (22.5) | 213 (17.1) |  |  |  | 177 (20.8) | 188 (22) |  |  |
| 250-499 | 2166 (35.6) | 174 (13.9) |  |  |  | 181 (21.2) | 161 (18.9) |  |  |
| ≥500 | 2491 (40.9) | 848 (67.9) |  |  |  | 490 (57.4) | 500 (58.6) |  |  |
| **Teaching, No. (%)** | 1144 (18.8) | 706 (56.5) | 0.846 | <0.001 |  | 354 (41.5) | 352 (41.3) | 0.005 | 0.961 |
| **Provider region, No. (%)** |  |  |  |  |  |  |  |  |  |
| Midwest | 1714 (28.1) | 267 (21.4) | 1.058 | <0.001 |  | 240 (28.1) | 263 (30.8) | 0.102 | 0.218 |
| Northeast | 658 (10.8) | 627 (50.2) |  |  |  | 264 (30.9) | 251 (29.4) |  |  |
| South | 1883 (30.9) | 279 (22.3) |  |  |  | 251 (29.4) | 263 (30.8) |  |  |
| West | 1834 (30.1) | 76 (6.1) |  |  |  | 98 (11.5) | 76 (8.9) |  |  |
| **Using first-line sedation or opioids drugs, No. (%)** |  |  |  |  |  |  |  |  |  |
| Dexmedetomidine | 1034 (17.0) | 192 (15.4) | 0.044 | 0.178 |  | 156 (18.3) | 162 (19.0) | 0.018 | 0.756 |
| Morphine | 209 (3.4) | 77 (6.2) | 0.128 | <0.001 |  | 39 (4.6) | 46 (5.4) | 0.038 | 0.504 |
| Fentanyl | 2472 (40.6) | 968 (77.5) | 0.810 | <0.001 |  | 555 (65.1) | 583 (68.3) | 0.070 | 0.165 |

Abbreviations: AaDO2, alveolar-arterial oxygen difference; APACHE-III score, the acute physiology and chronic health evaluation III score; CCU, cardiac care unit; Dex, dexmedetomidine; NICU, neurological intensive care unit; SICU, surgical intensive care unit.

* Data shown as mean ± standard deviation, number (percent), or median (interquartile range) as appropriate.

# SD = standardized difference (SD ≥ 0.1 represent significant differences in covariables between groups).

**Additional file 10. Demographics and clinical characteristics of the patients who received midazolam or dexmedetomidine before and after propensity score adjustment**

| **Clinical Variable*** | **Baseline covariates of patient  before Propensity Matching** | | | |  | **Baseline covariates of patient  after Propensity Matching** | | | |
| --- | --- | --- | --- | --- | --- | --- | --- | --- | --- |
|  | **Midazolam (N=2,051)** | **Dex (N=1,566)** | **SD^#^** | ***P* value** |  | **Midazolam (N=816)** | **Dex (N=816)** | **SD** | ***P* value** |
| **Age, No. (%)** |  |  |  |  |  |  |  |  |  |
| 18 to 45 years | 219 (14.0) | 356 (17.4) | 0.152 | <0.001 |  | 122 (15.0) | 132 (16.2) | 0.045 | 0.932 |
| 46 to 65 years | 581 (37.1) | 835 (40.7) |  |  |  | 318 (39.0) | 309 (37.9) |  |  |
| 66 to 80 years | 574 (36.7) | 635 (31.0) |  |  |  | 277 (33.9) | 283 (34.7) |  |  |
| 81 to 89 years | 170 (10.9) | 191 (9.3) |  |  |  | 85 (10.4) | 79 (9.7) |  |  |
| over 89 years | 22 (1.4) | 34 (1.7) |  |  |  | 14 (1.7) | 13 (1.6) |  |  |
| **Female, No. (%)** | 635 (40.5) | 871 (42.5) | 0.039 | 0.26 |  | 334 (40.9) | 338 (41.4) | 0.010 | 0.880 |
| **Ethnicity, No. (%)** |  |  |  |  |  |  |  |  |  |
| White | 1118 (71.4) | 1590 (77.5) | 0.275 | <0.001 |  | 600 (73.5) | 587 (71.9) | 0.065 | 0.789 |
| Black | 145 (9.3) | 193 (9.4) |  |  |  | 84 (10.3) | 94 (11.5) |  |  |
| Latino | 191 (12.2) | 98 (4.8) |  |  |  | 72 (8.8) | 71 (8.7) |  |  |
| Asian | 10 (0.6) | 26 (1.3) |  |  |  | 6 (0.7) | 10 (1.2) |  |  |
| Other | 102 (6.5) | 144 (7) |  |  |  | 54 (6.6) | 54 (6.6) |  |  |
| **Weight, kg** | 81.1 (67.6-97.5) | 83 (67.1-100) | 0.075 | 0.026 |  | 80.6 (67.3-99) | 82.2 (66.9-99.9) | 0.004 | 0.942 |
| **Height, cm** | 170.2 (162.5-177.8) | 170.2 (162.6-177.8) | 0.020 | 0.553 |  | 170.2 (162.5-177.9) | 170.2 (162.6-177.8) | 0.009 | 0.852 |
| **APACHE-III score** | 53 (38.0-71.0) | 63 (43.0-85.0) | 0.351 | <0.001 |  | 60 (43.0-79.0) | 58 (41.0-82.0) | 0.037 | 0.459 |
| **Oxgenation index, mmHg** | 150 (90.0-201.4) | 130 (66.3-192.5) | 0.187 | <0.001 |  | 140.3 (86.0-195.6) | 148.4 (84.0-201.8) | 0.004 | 0.941 |
| **AaDo2, mmHg** | 321.1 (213.7-517.6) | 360.5 (243.4-560.2) | 0.175 | <0.001 |  | 340.1 (223.5-532.8) | 339.9 (226.2-546.3) | <0.001 | 0.997 |
| **Patient characteristics at discharge, No. (%)** |  |  |  |  |  |  |  |  |  |
| ARDS | 1182 (75.5) | 1693 (82.5) | 0.174 | <0.001 |  | 615 (75.4) | 607 (74.4) | 0.023 | 0.690 |
| Pneumonia | 342 (21.8) | 771 (37.6) | 0.350 | <0.001 |  | 207 (25.4) | 202 (24.8) | 0.014 | 0.819 |
| Sepsis | 317 (20.2) | 728 (35.5) | 0.345 | <0.001 |  | 209 (25.6) | 215 (26.3) | 0.017 | 0.778 |
| Aspiration | 149 (9.5) | 278 (13.6) | 0.127 | <0.001 |  | 80 (9.8) | 80 (9.8) | <0.001 | 1.000 |
| Heart failure | 88 (5.6) | 182 (8.9) | 0.126 | <0.001 |  | 45 (5.5) | 51 (6.2) | 0.031 | 0.599 |
| Chronic pulmonary disease | 202 (12.9) | 360 (17.6) | 0.130 | <0.001 |  | 112 (13.7) | 111 (13.6) | 0.004 | 1.000 |
| Liver disease | 16 (1.0) | 91 (4.4) | 0.211 | <0.001 |  | 14 (1.7) | 13 (1.6) | 0.010 | 1.000 |
| Renal failure | 124 (7.9) | 255 (12.4) | 0.150 | <0.001 |  | 66 (8.1) | 64 (7.8) | 0.009 | 0.927 |
| Hypertension | 241 (15.4) | 404 (19.7) | 0.113 | 0.001 |  | 98 (12) | 90 (11) | 0.031 | 0.587 |
| Diabetes | 68 (4.3) | 139 (6.8) | 0.106 | 0.002 |  | 29 (3.6) | 30 (3.7) | 0.007 | 1.000 |
| **Hospital characteristics** |  |  |  |  |  |  |  |  |  |
| **ICU type, No. (%)** |  |  |  |  |  |  |  |  |  |
| SICU | 98 (6.3) | 293 (14.3) | 0.294 | <0.001 |  | 53 (6.5) | 48 (5.9) | 0.047 | 0.827 |
| CCU | 348 (22.2) | 383 (18.7) |  |  |  | 152 (18.6) | 163 (20) |  |  |
| NICU | 91 (5.8) | 66 (3.2) |  |  |  | 44 (5.4) | 48 (5.9) |  |  |
| Others | 1029 (65.7) | 1309 (63.8) |  |  |  | 567 (69.5) | 557 (68.3) |  |  |
| **Number of beds, No. (%)** |  |  |  |  |  |  |  |  |  |
| <100 | 0 (0.0) | 16 (0.8) | 0.209 | <0.001 |  | 0 (0.0) | 0 (0.0) | 0.071 | 0.363 |
| 100-249 | 250 (16) | 343 (16.7) |  |  |  | 176 (21.6) | 199 (24.4) |  |  |
| 250-499 | 397 (25.4) | 376 (18.3) |  |  |  | 235 (28.8) | 234 (28.7) |  |  |
| ≥500 | 919 (58.7) | 1316 (64.2) |  |  |  | 405 (49.6) | 383 (46.9) |  |  |
| **Teaching, No. (%)** | 258 (16.5) | 1070 (52.2) | 0.811 | <0.001 |  | 171 (21) | 167 (20.5) | 0.012 | 0.855 |
| **Provider region, No. (%)** |  |  |  |  |  |  |  |  |  |
| Midwest | 681 (43.5) | 391 (19.1) | 1.264 | <0.001 |  | 323 (39.6) | 322 (39.5) | 0.041 | 0.880 |
| Northeast | 49 (3.1) | 1026 (50) |  |  |  | 49 (6) | 42 (5.1) |  |  |
| South | 645 (41.2) | 464 (22.6) |  |  |  | 317 (38.8) | 326 (40) |  |  |
| West | 191 (12.2) | 170 (8.3) |  |  |  | 127 (15.6) | 126 (15.4) |  |  |
| **Using first-line sedation or opioids drugs, No. (%)** |  |  |  |  |  |  |  |  |  |
| Propofol | 1034 (66) | 994 (48.5) | 0.361 | <0.001 |  | 520 (63.7) | 491 (60.2) | 0.073 | 0.153 |
| Morphine | 45 (2.9) | 205 (10) | 0.293 | <0.001 |  | 33 (4.0) | 34 (4.2) | 0.006 | 1.000 |
| Fentanyl | 641 (40.9) | 1570 (76.5) | 0.776 | <0.001 |  | 488 (59.8) | 512 (62.7) | <0.001 | 0.242 |

Abbreviations: AaDO2, alveolar-arterial oxygen difference; APACHE-III score, the acute physiology and chronic health evaluation III score; CCU, cardiac care unit; Dex, dexmedetomidine; NICU, neurological intensive care unit; SICU, surgical intensive care unit.

* Data shown as mean ± standard deviation, number (percent), or median (interquartile range) as appropriate.

# SD = standardized difference (SD ≥ 0.1 represent significant differences in covariables between groups).

**Additional file 11. Demographics and clinical characteristics of the patients who received propofol or dexmedetomidine before and after propensity score adjustment**

| **Clinical Variable*** | **Baseline covariates of patient  before Propensity Matching** | | | |  | **Baseline covariates of patient  after Propensity Matching** | | | |
| --- | --- | --- | --- | --- | --- | --- | --- | --- | --- |
|  | **Dex (N=724)** | **Propofol (N=6,049)** | **SD^#^** | ***P* value** |  | **Dex (N=678)** | **Propofol (N=678)** | **SD** | ***P* value** |
| **Age, No. (%)** |  |  |  |  |  |  |  |  |  |
| 18 to 45 years | 89 (12.3) | 947 (15.7) | 0.104 | 0.159 |  | 81 (11.9) | 76 (11.2) | 0.073 | 0.769 |
| 46 to 65 years | 288 (39.8) | 2398 (39.6) |  |  |  | 270 (39.8) | 290 (42.8) |  |  |
| 66 to 80 years | 251 (34.7) | 1926 (31.8) |  |  |  | 234 (34.5) | 215 (31.7) |  |  |
| 81 to 89 years | 80 (11) | 659 (10.9) |  |  |  | 77 (11.4) | 79 (11.7) |  |  |
| over 89 years | 16 (2.2) | 119 (2) |  |  |  | 16 (2.4) | 18 (2.7) |  |  |
| **Female, No. (%)** | 436 (60.2) | 3387 (56) | 0.086 | 0.033 |  | 406 (59.9) | 415 (61.2) | 0.027 | 0.657 |
| **Ethnicity, No. (%)** |  |  |  |  |  |  |  |  |  |
| White | 540 (74.6) | 4610 (76.2) | 0.122 | 0.047 |  | 503 (74.2) | 508 (74.9) | 0.070 | 0.796 |
| Black | 64 (8.8) | 554 (9.2) |  |  |  | 63 (9.3) | 54 (8) |  |  |
| Latino | 67 (9.3) | 399 (6.6) |  |  |  | 61 (9) | 56 (8.3) |  |  |
| Asian | 4 (0.6) | 76 (1.3) |  |  |  | 4 (0.6) | 5 (0.7) |  |  |
| Other | 49 (6.8) | 410 (6.8) |  |  |  | 47 (6.9) | 55 (8.1) |  |  |
| **Weight, kg** | 80.7 (65-97.1) | 81.2 (67.6-99.4) | 0.045 | 0.242 |  | 80.6 (65.5-98.7) | 81.6 (68.1-99) | 0.016 | 0.770 |
| **Height, cm** | 170.2 (162.5-177.8) | 170.2 (162.6-177.8) | 0.022 | 0.577 |  | 170.2 (162.5-177.8) | 170.2 (162.6-177.8) | 0.033 | 0.544 |
| **APACHE-III score** | 54 (37-76) | 54 (38-74) | 0.003 | 0.939 |  | 55 (38-76) | 52 (37-72) | 0.058 | 0.286 |
| **Oxgenation index, mmHg** | 157 (93.3-204.3) | 168.2 (106-223.3) | 0.122 | 0.002 |  | 156.1 (93.2-208.2) | 157.9 (96.8-220.8) | 0.052 | 0.342 |
| **AaDo2, mmHg** | 308.6 (185.9-522.8) | 297.2 (209.6-472.1) | 0.069 | 0.070 |  | 308.6 (188.8-524) | 298.3 (199.6-469.3) | 0.071 | 0.192 |
| **Patient characteristics at discharge, No. (%)** |  |  |  |  |  |  |  |  |  |
| ARDS | 510 (70.4) | 4564 (75.5) | 0.113 | 0.004 |  | 482 (71.1) | 482 (71.1) | <0.001 | 1.000 |
| Pneumonia | 194 (26.8) | 1474 (24.4) | 0.056 | 0.165 |  | 178 (26.3) | 171 (25.2) | 0.024 | 0.709 |
| Sepsis | 153 (21.1) | 1588 (26.3) | 0.121 | 0.003 |  | 148 (21.8) | 142 (20.9) | 0.022 | 0.741 |
| Aspiration | 71 (9.8) | 621 (10.3) | 0.015 | 0.748 |  | 63 (9.3) | 66 (9.7) | 0.015 | 0.853 |
| Heart failure | 33 (4.6) | 411 (6.8) | 0.097 | 0.027 |  | 32 (4.7) | 27 (4) | 0.036 | 0.594 |
| Chronic pulmonary disease | 111 (15.3) | 875 (14.5) | 0.024 | 0.569 |  | 104 (15.3) | 117 (17.3) | 0.052 | 0.378 |
| Liver disease | 16 (2.2) | 135 (2.2) | 0.001 | 1.000 |  | 15 (2.2) | 16 (2.4) | 0.010 | 1.000 |
| Renal failure | 53 (7.3) | 759 (12.5) | 0.175 | 0.269 |  | 53 (7.8) | 46 (6.8) | 0.004 | 0.531 |
| Hypertension | 104 (14.4) | 1173 (19.4) | 0.135 | 0.001 |  | 96 (14.2) | 84 (12.4) | 0.052 | 0.379 |
| Diabetes | 30 (4.1) | 222 (3.7) | 0.024 | 0.594 |  | 30 (4.4) | 30 (4.4) | <0.001 | 1.000 |
| **Hospital characteristics** |  |  |  |  |  |  |  |  |  |
| **ICU type, No. (%)** |  |  |  |  |  |  |  |  |  |
| SICU | 60 (8.3) | 601 (9.9) | 0.175 | <0.001 |  | 56 (8.3) | 44 (6.5) | 0.095 | 0.387 |
| CCU | 184 (25.4) | 1121 (18.5) |  |  |  | 162 (23.9) | 175 (25.8) |  |  |
| NICU | 37 (5.1) | 276 (4.6) |  |  |  | 35 (5.2) | 27 (4) |  |  |
| Others | 443 (61.2) | 4051 (67) |  |  |  | 425 (62.7) | 432 (63.7) |  |  |
| **Number of beds, No. (%)** |  |  |  |  |  |  |  |  |  |
| <100 | 2 (0.3) | 65 (1.1) | 0.408 | <0.001 |  | 2 (0.3) | 2 (0.3) | 0.082 | 0.516 |
| 100-249 | 138 (19.1) | 1389 (23) |  |  |  | 138 (20.4) | 155 (22.9) |  |  |
| 250-499 | 151 (20.9) | 2122 (35.1) |  |  |  | 151 (22.3) | 161 (23.7) |  |  |
| ≥500 | 433 (59.8) | 2473 (40.9) |  |  |  | 387 (57.1) | 360 (53.1) |  |  |
| **Teaching, No. (%)** | 174 (24) | 1424 (23.5) | 0.012 | 0.804 |  | 158 (23.3) | 147 (21.7) | 0.039 | 0.515 |
| **Provider region, No. (%)** |  |  |  |  |  |  |  |  |  |
| Midwest | 302 (41.7) | 1459 (24.1) | 0.697 | <0.001 |  | 277 (40.9) | 283 (41.7) | 0.032 | 0.952 |
| Northeast | 48 (6.6) | 1056 (17.5) |  |  |  | 48 (7.1) | 43 (6.3) |  |  |
| South | 300 (41.4) | 1723 (28.5) |  |  |  | 279 (41.2) | 278 (41) |  |  |
| West | 74 (10.2) | 1811 (29.9) |  |  |  | 74 (10.9) | 74 (10.9) |  |  |
| **Using first-line sedation or opioids drugs, No. (%)** |  |  |  |  |  |  |  |  |  |
| Midazolam | 192 (26.5) | 994 (16.4) | 0.248 | <0.001 |  | 168 (24.8) | 164 (24.2) | 0.014 | 0.850 |
| Morphine | 20 (2.8) | 312 (5.2) | 0.123 | 0.006 |  | 20 (2.9) | 15 (2.2) | 0.047 | 0.493 |
| Fentanyl | 307 (42.4) | 2740 (45.3) | 0.058 | 0.150 |  | 286 (42.2) | 307 (45.3) | 0.062 | 0.274 |

Abbreviations: AaDO2, alveolar-arterial oxygen difference; APACHE-III score, the acute physiology and chronic health evaluation III score; CCU, cardiac care unit; Dex, dexmedetomidine; NICU, neurological intensive care unit; SICU, surgical intensive care unit.

* Data shown as mean ± standard deviation, number (percent), or median (interquartile range) as appropriate.

# SD = standardized difference (SD ≥ 0.1 represent significant differences in covariables between groups).
